# Predictive models for stage and risk classification in head and neck squamous cell carcinoma (HNSCC)

**DOI:** 10.7717/peerj.9656

**Published:** 2020-09-22

**Authors:** Sugandh Kumar, Srinivas Patnaik, Anshuman Dixit

**Affiliations:** 1 Computational Biology and Bioinformatics Laboratory, Institute of Life Science, Bhubaneswar, Odisha, India; 2 School of Biotechnology, Kalinga Institute of Industrial Technology (KIIT) University, Bhubaneswar, Odisha, India

**Keywords:** Head and neck cancer, TNM stage, Machine learning, Biomarker, microRNA, mRNA

## Abstract

Machine learning techniques are increasingly used in the analysis of high throughput genome sequencing data to better understand the disease process and design of therapeutic modalities. In the current study, we have applied state of the art machine learning (ML) algorithms (Random Forest (RF), Support Vector Machine Radial Kernel (svmR), Adaptive Boost (AdaBoost), averaged Neural Network (avNNet), and Gradient Boosting Machine (GBM)) to stratify the HNSCC patients in early and late clinical stages (TNM) and to predict the risk using miRNAs expression profiles. A six miRNA signature was identified that can stratify patients in the early and late stages. The mean accuracy, sensitivity, specificity, and area under the curve (AUC) was found to be 0.84, 0.87, 0.78, and 0.82, respectively indicating the robust performance of the generated model. The prognostic signature of eight miRNAs was identified using LASSO (least absolute shrinkage and selection operator) penalized regression. These miRNAs were found to be significantly associated with overall survival of the patients. The pathway and functional enrichment analysis of the identified biomarkers revealed their involvement in important cancer pathways such as GP6 signalling, Wnt signalling, p53 signalling, granulocyte adhesion, and dipedesis. To the best of our knowledge, this is the first such study and we hope that these signature miRNAs will be useful for the risk stratification of patients and the design of therapeutic modalities.

## Introduction

Head and neck squamous cell carcinoma (HNSCC) is the 6th most common type of cancer (Bray et al., 2018) and is associated with 650,000 new cases and ∼330,000 deaths annually worldwide (Boscolo-Rizzo et al., 2018; Kapoor & Kumar, 2019). The majority of the HNSCC cases are Oral Squamous Cell Carcinomas (OSCC) (Deshpande & Wong, 2008). More than 90% of HNSCC are associated with OSCC patients (Vigneswaran & Williams, 2014). The incidence rates (mainly OSCC (Collaboration, 2019)) are higher in South Asian countries such as India (Poddar et al., 2019), Bangladesh (Collaboration, 2019), and Pakistan (Akhtar et al., 2016) as compared to other parts of the world. There are several known risk factors of HNSCC such as chewing tobacco, smoking cigarettes, excessive alcohol consumption (Stenson, Brockstein & Ross, 2016) and oncogenic virus such as Human papillomavirus (HPV) (Marur et al., 2010). Additionally, epigenetic regulation, mutation, copy number variation (CNV) and immune host response also play a key role in carcinogenesis (Network, 2015; Leemans, Snijders & Brakenhoff, 2018). Despite current advancement in cancer diagnosis and treatment, the overall 5-year survival rate is less than ∼50% in HNSCC due to a lack of proper diagnostic markers and targeted therapies (Wiegand et al., 2015).

It has been well documented that detection in early stages leads to higher survival as compared to late stages in various cancers including HNSCC. The American Joint Committee of Cancer staging (TNM) describes an early stage, primary tumor as ∼2–4 cm in diameter without lymph node proliferation and metastasis (TNM stage I and II). The tumor is considered advanced (late stage), if it is larger (>5 cm) and has spread into either nearby lymph nodes only (TNM stage III) or has metastasized to other parts of the body also (TNM stage IV) (https://www.cancer.org/treatment/understanding-your-diagnosis/staging.html).

A lot of new research have happened in HNSCC in the past few decades, however without clinically meaningful discoveries. While there are some biomarkers (e.g., HPV +ve and −ve), however, they lack important features, such as high specificity and sensitivity, low cost, and short turnaround time. A quick and accurate diagnosis would have numerous benefits for the patients such as proper treatment resulting in reduced morbidity as well as and improve treatment outcomes. Unfortunately, there is no such universally accepted biomarker for HNSCC accepted for clinical use. There is an urgent need for more effective therapies, and clinically relevant biomarkers to stratify patients in HNSCC.

The micro-RNAs (miRNAs) are ∼18–25 nucleotide long non-coding RNAs. They can regulate mRNA expression by interacting with the 3′ untranslated regions (UTR) leading to mRNA degradation. These miRNAs by virtue of their control over mRNA expression have important regulatory roles such as regulation of cell division, cell maturation, angiogenesis, proliferation, migration, invasion, metastasis, autophagy, and apoptosis (MacFarlane & Murphy, 2010). However, in various diseases especially cancers, these miRNAs themselves can get dysregulated leading to pathological conditions (Esteller, 2011). A large number of miRNAs have been quite well characterized for their biological function in cancer and their ability to regulate the expression of different cancer pathways (Iorio & Croce, 2012). It is also known that the changes in miRNA expression profile can be detected even before the appearance of clinical symptoms in some cancers (Bianchi et al., 2011). These miRNA due to their stability and ease of detection (in tissues as well as biological fluids) offer a rational approach for the development of excellent biomarkers (Mostert et al., 2011). The analysis of miRNA expression profiles may also offer an insight into underlying tumor progression and/or identifying new therapeutic targets.

Mathematical modeling has been widely used in disease modeling, classification and molecular function prediction for long. The advancement of next generation sequencing (NGS) technology and the availability of massive sequencing data have opened new avenues in disease process understanding and monitoring by machine learning. The machine learning techniques are generally used for risk stratification, mutational frequency prediction, CNV, and new target identification. It has been suggested that machine learning (ML) techniques can be utilized for diagnosis and prognosis in cancers (Obermeyer & Emanuel, 2016). For instance, the ML techniques have been used for the detection of Rat Sarcoma (RAS) activation pathways in cancers utilizing expression data, SNPs (single nucleotide polymorphisms) and CNVs (copy number variations) (Way et al., 2018). A multi-parametric Decision Support System (DSS) with a multitude of heterogenic data (clinical information, genomics, and imaging data) has been used to predict the OSCC progression and potential relapse (local or metastatic) (Komura & Ishikawa, 2018). A study by Avissar et al. (2009) for identification of the miRNAs to predict the presence of HNSCC identified miR-221 and miR375 to be predictors. However, the study was done without considering stages/grade. Recently, a machine learning study used neural networks to predict recurrences in tongue cancer (Alabi et al., 2019). Kim et al. (2019) developed predictive models for survival prediction in oral cancer patients. There is no report to date that stratifies the clinical stages in HNSCC patients using miRNA expression profiles. The identification of late stages often involves cumbersome examination and invasive tests; therefore, such studies could be immensely useful in accomplishing better diagnosis and clinical outcomes. Such strategy can also be extended to the cancers that are difficult to reach and detect such as pancreatic cancer.

In the current study, we have analyzed the miRNA expression patterns in HNSCC patients in order to identify miRNA signatures that can distinguish stages (early and late stage) using machine learning approaches. It has to be remembered that the clinical stage is known to play an important role in the overall survival of the patients. These studies have led to the identification of signatures that can efficiently stratify the HNSCC patients into early and late clinical stages as well as to stratify patient’s risk that may help in the decision of the treatment regimens e.g., whether it is effective to use less aggressive treatment than highly toxic therapies.

## Materials and Methods

**Figure 1:**
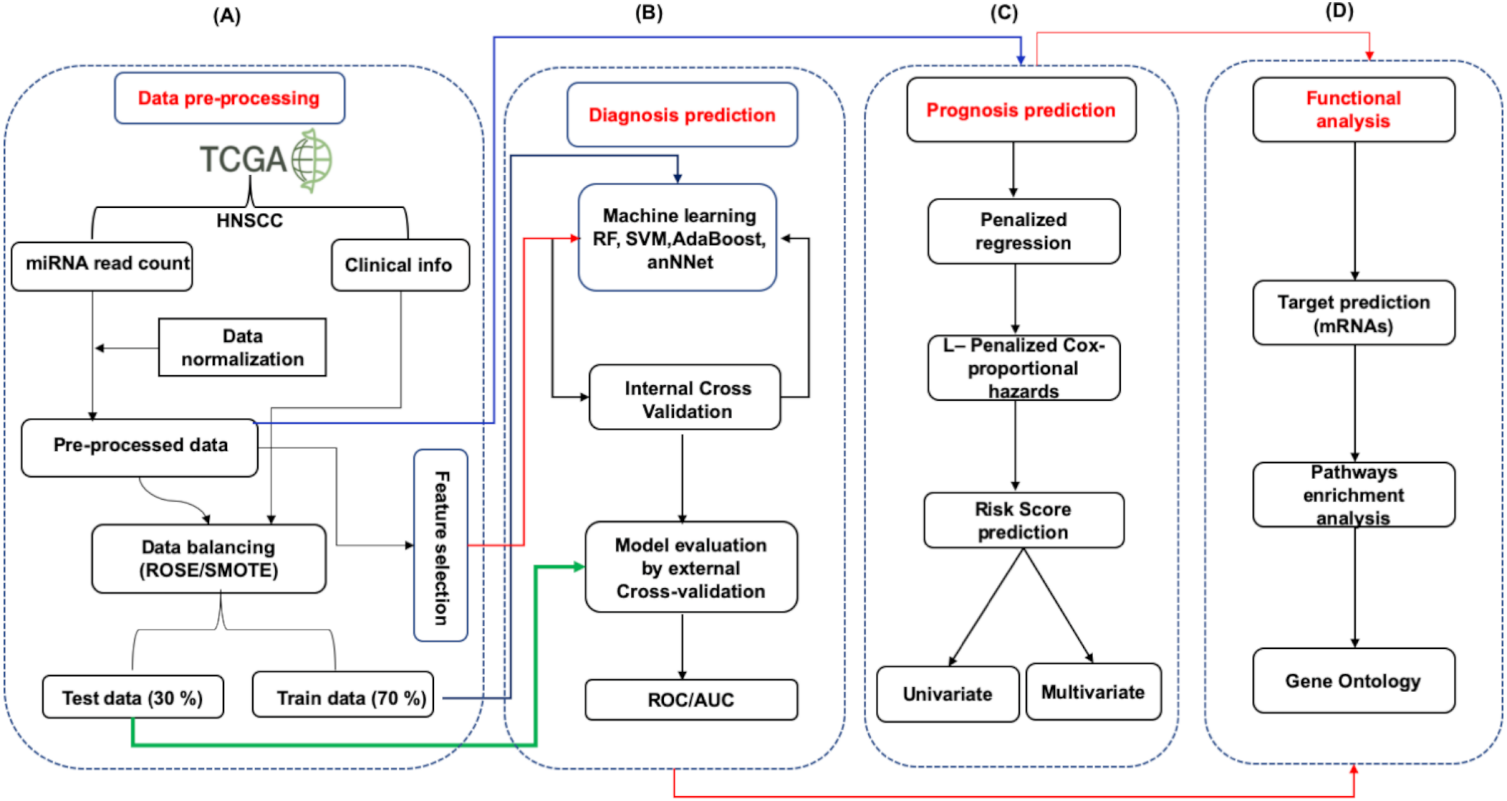
The schematic representation of workflow. The study was divided into four parts. (A) The expression data (mRNA and miRNA) along with clinical information of HNSCC patients was obtained from the TCGA and was preprocessed by *Z*-score normalization and balancing (ROSE/SMOTE) prior to model building. The data was then systematically divided into training (70%) and test (30%) sets. (B) Identification of signature miRNA for early and late stage classification was accomplished using the miRNA expression data. The training set was used for feature selection and model building using machine learning techniques viz. Random Forest (RF), Support Vector Machine using radial kernel (svmR), adaptive boosting (adaBoost), Average neural network (avNNet), and Gradient Boosting Machine (GBM). The test set was used as an external test set to rigorously and independently assess the performance of the generated model. (C) Prognostic miRNA signature was identified using the LASSO (least absolute shrinkage and selection operator) based L1-Cox-proportional hazards method. The individual miRNA was then accessed for their influence on the overall survival of the patients using the Kaplan-Meir test. (D) The functional analysis involved mRNA target prediction, pathways analysis, and Gene Ontology (GO).

The overall strategy involves the following steps. (1) data processing (2) data distribution into training (70%) and test set (30%) (3) model building (4) 10-fold repeated internal cross validation (5) independent external cross-validation. The detailed workflow has been provided in Fig. 1. The studies were divided into three parts (i) Identification of diagnostic signature: Involving the identification of signature miRNAs for patient’s classification into early and late stage (ii) Identification of prognostic signature: evaluation of independent prognostic marker using L1-Cox Proportional Hazards Model with penalized regression to predict the risk to the patient (iii) Functional analysis: The identification of pathways and processes regulated/influenced by the identified biomarkers.

### Data retrieval and pre-processing

The normalized reads of miRNAs (Illumina HiSeq 2000 platform, miRgene-level, Normalized), mRNAs (llumina HiSeq 2000 platform, Gene-level) expression were retrieved from the TCGA (https://cancergenome.nih.gov). A total of 528 samples (484 primary tumors and 44 Normal Adjacent Tissue (NAT)) with associated clinical information such as TNM stage, status, gender, and age etc. (Table S1) were selected whereas the samples with unknown stage information were removed. Finally, the data contained 27, 74, 78 and 274 samples from stage I–IV respectively (total 453 samples). The stages I and II were treated as early stage (total 101 samples) and stage III and IV as later stage (total 352 samples). Further, miRNAs and mRNAs expression profiles were normalized using the *limma-voom* (Law et al., 2014) library package in R3.3.5.

### Differential miRNA and mRNAs expression analysis

Differential expression (DE) analysis of miRNAs and mRNAs were performed by the limma-voom using student’s *t*-test. The mRNAs and miRNAs were considered differentially expressed if (|log_2_FC ≥ 1| and *p*-value < 0.05). The volcano plot and heat map were generated using Enhancevolcano (Blighe, 2018) and Complexheatmap (Gu, Eils & Schlesner, 2016) library in *R3.3.5*.

### Data scaling

In the current study the data was scaled using *Z-scaling*. The following equation was used for the scaling of data set.

(1) \begin{eqnarray*}& & {x}^{{^{\prime}}}= \frac{x-\overline{x}}{\sigma } .\end{eqnarray*}

Here, $x-\overline{x}$, represented the expression variance. While *σ* is the standard deviation of miRNAs expression. The *x*′ is the normalized miRNAs expression. The ‘*Z-score*’ method represent that data scaled to a mean of zero and a standard deviation of 1.

### ML model building for stage classification

The *“caret”* library of R package (Kuhn, 2012) that contains several machine learning, complex regression and classification methods was utilized to develop various ML algorithm based classification models. The machine learning techniques were employed for building the models for patient classification into clinical stages using miRNA expression profiles. In this study, four widely used machine learning (ML) methods namely RF, svmR, AdaBoost, and avNNet were used.

The random forest (RF) method commonly uses non-linear regression models and has been applied in a variety of computational studies. It is a simple, interpretive, and flexible method which allows for a large number of predictor variables. Additionally, it also predicts well in the small sample sizes and high genetic heterogeneity (Alexopoulos, 2010). It comprises building trees from bootstrapped training data in such a way that each split is based on a random sample of *m* variables from a full set of *n* attributes (Liaw & Wiener, 2002).

(2) \begin{eqnarray*}& & IG(n)=1-\sum _{i=1}^{j}(pi)^{2}.\end{eqnarray*}

Here IG is Gini impurity of a node *n* is 1- the sum of overall predictor as *j* of the fraction of each *pi* square

Support Vector Machine (SVM) is popular ML technique in medical research for molecular function prediction, genomics variation, histopathology image classifications and subtyping of diseases (Bianchi et al., 2011). It is a non-probabilistic algorithm classifier based on the hyperplane line to maximize the maximum margin to separate two classes based on the two vectors points to achieve the best fit classification (Huang et al., 2018).

(3) \begin{eqnarray*}& & \int \nolimits \left( x \right) ={\beta }_{0}+\sum _{i\in S}{\alpha }_{i}K(x,{x}_{i}).\end{eqnarray*}

Here, function (*x*) is the kernel function which calculate the similarity between training and predicted *x*, *x*_*i*_ and *α*_*i*_ represent the parameter corresponding to each training and predictor variable. *β*_0_ isthe constant.

The AdaBoost is an algorithm for producing strong classifiers from the weak classifier. It is based on the penalized weighted matrix of each instance and voting them according to their weight scheme. It is a popular machine learning method for both balance and unbalanced data sets (Zemel & Pitassi, 2001). The AdaBoost equation is following

(4) \begin{eqnarray*}& & H(x)=\mathrm{sign} \left( \sum _{t=1}^{T}{\alpha }_{t}{h}_{t}(x) \right) .\end{eqnarray*}

Where, *h*_*t*_(*x*) is the out of weak classifier of *t* for input *x* and *α*_*t*_ is weight given to model. The *α*_*t*_ = 0.5∗ln(1 − *E*)∕*E*. Here, *E* is based on the error rate of model.

The avNNeT is a kind of neural network capable of learning nearly infinite number of mapping functions. The neural network (NN) behaves like a natural human neuron. Every predictor variables (inputs) connects to an output response variable (output), either directly or through backward and forward propagation of single and several of hidden nodes to calculate units (neurons). The depth of an NN corresponds to the number of hidden layers. The calculation performed in NN are performed in a hidden layer called deep network (Huang, Zhu & Siew, 2004). The NN is commonly used for calculation of gene expression, CNV and clinical data for prognosis prediction of complex diseases such as cancer (Mirza et al., 2019).

(5) \begin{eqnarray*}{a}_{j}^{l}=\sigma \left( \sum _{k}{w}_{jk}^{l}{a}_{k}^{l-1}+{b}_{j}^{l} \right) .\end{eqnarray*}

Here, ${a}_{j}^{l}$ of the *j*th neuron in the *l*th layer is related to the activation in the (*l* − 1)th layer. The *σ* is vectorising function. The *W*^*l*^ is weight matrix of each layer, *l.* The *j*th row and *k*th column is ${w}_{jk}^{l}$ . Also, for each layer *l* as define *j* a bias vector ${b}_{j}^{l}$.

The GBM algorithm is used to convert weak learners into strong learners. It is the tree-decision boosting based classification model where each of instances assigned an equal weight at the start. After the examination of first steps, then increase the weight of those observation that are difficult to classify and lower the weight for those that are easy to classify. This is the iterative process to optimized the best fit for classification (Friedman, 2001). The general equation used for the GBM is given below

(6) \begin{eqnarray*}{h}_{m} \left( x \right) =\sum _{j=1}^{{j}_{m}}{b}_{jm}1{R}_{jm} \left( x \right) .\end{eqnarray*}

Here, m-th step fit the decision tree hm (x). jm number leaves in each steps. The *b*_*jm*_ is the value predicted in the region of *R*_*jm*_.

*Data balancing*: A challenge in biomedical studies is working with imbalanced data sets i.e., unequal number of normal and disease samples. Unbalanced predictive variable ratio do not meet the assumptions of the machine learning models and its predict biased. Therefore, SMOTE algorithm (Chawla et al., 2002) was used to balance the data using the ‘DMwR’ library in R.3.3.5 package. The SMOTE is a popular algorithm to deal with unbalanced data (Lusa, 2013; Ramezankhani et al., 2016). There are two types of sampling strategies commonly employed in machine learning viz. oversampling and under-sampling for unbiased classification. In under-sampling, the samples are reduced based on k nearest neighbor (kNN) clustering centroid distance while in oversampling minority classes are amplified to balance both predictors. In our data set, the number of late stage patient samples was reduced so as to match the number of early stage patient samples (*N* = 101) (Fig. S1A) using the under-sampling method and in oversampling (Fig. S1B), early stage data was amplified (*N* = 352) to balanced early and later-stage samples.

Thereafter, the datasets (under and over sampled) were systematically distributed into training (70%) and test set (30%) separately. In under-sampled data, 141 and 61 samples were used as training and test set respectively whereas in oversampled data 478 and 206 samples were used as training and test set respectively. First, the model was trained using only training set the test set was kept aside for independent external cross-validation. A 10-fold repeated internal cross-validation with 10-time iterations to randomized data set was done to avoid generation of over/under fitting model. The cost functions were optimized (100 to 3,000 with 100 steps per iteration) to achieve accurate classification. The performance of the generated model was examined based on sensitivity, specificity, accuracy, and AUC (ROC), precision and MCC by external independent data. Equations are given below:

(7) \begin{eqnarray*}& & \text{Sensitivity}= \frac{\mathrm{TP}}{\mathrm{TP}+\mathrm{FN}} \end{eqnarray*}

(8) \begin{eqnarray*}& & \text{Specificity}= \frac{\mathrm{TN}}{\mathrm{TP}+\mathrm{FP}} \end{eqnarray*}

(9) \begin{eqnarray*}& & \text{Precision}= \frac{\mathrm{TP}}{\mathrm{TP}+\mathrm{FP}} \end{eqnarray*}

(10) \begin{eqnarray*}& & \mathrm{MCC}= \frac{\mathrm{TP}\times \mathrm{TN}-\mathrm{FP}\times \mathrm{FN}}{\sqrt{ \left( \mathrm{TP}+\mathrm{FP} \right) \left( \mathrm{TP}+\mathrm{FN} \right) \left( \mathrm{TN}+\mathrm{FP} \right) (\mathrm{TN}+\mathrm{FN})}} .\end{eqnarray*}

Where TP is true positive; TN is true negative; FP false positive; and FN is false negative, MCC is Matthews correlation coefficient.

### Patient’s risk assessment based on signature miRNA expression

Further, we performed L-1 Penalized Estimation in the Cox Proportional Hazards Model using optimal cross-validated likelihood to identify prognostic markers based on the survival status (alive/dead), survival time and normalized miRNAs expression (Acharya et al., 2017). The Kaplan–Meier curve and the Log-rank method were used to estimate the significance of median miRNA expression on the survival of the patients. It evaluates the independent survival of the patient between the two risk groups (low/high). The patient’s clinical parameters such as age, survival status, gender, metastasis, TNM stages, and median expression of significant miRNAs were evaluated for their association with patient’s survival using cox-regression for a univariate and multivariate analysis.

The risk score for individual patients was calculated using L1-penalized (LASSO) likelihood Cox-regression (Goeman, 2010). As earlier, the data was partitioned into training (70%) and test (30%) to assess the performance of the generated models. The prognostic value of individual miRNAs was first calculated by the L1-penalised Cox univariate proportional hazard regression in R tool using a ‘penalized’ package. The significant miRNAs (*p*-value < 0.05) were used in subsequent Cox multivariate analysis. The prognostic model was built by the linear combination of the expression level of significant miRNAs multiplied with the Cox regression coefficient (*β*). The standard formula was as follows:

(11) \begin{eqnarray*}& & \text{Risk Score}={X}_{1}{\beta }_{1}+{X}_{2}{\beta }_{2}+\cdots +{X}_{n}\beta n+C.\end{eqnarray*}

Using the median risk score the patients were divided into high- and low-risk groups. The time-dependent receiver-operating characteristic (ROC) curve was created using “survivalROC” package in R to evaluate the performance (specificity and sensitivity) of the generated miRNA prognostic signature (Chawla et al., 2002).

### The mRNAs target identification for identified miRNAs

The mRNAs targets of the identified biomarkers (diagnostic and prognostic) were predicted using the IPA (Ingenuity Pathway Analysis). The IPA predict miRNAs target based on three evidence (i) experimental (ii) high confidence (Kozomara & Griffiths-Jones, 2014) and (iii) low confidence. The experimental and high confidence interactions are curated by experts. In the current studies only experimentally validated and high confidence miRNA-mRNA interactions were used. The miRNAs can have a broad range of mRNAs targets and many of them may not be relevant to the disease under consideration. Therefore, the predicted target mRNAs were considered only if they are also significantly differentially expressed in the HNSCC. As a result, 2 sets (set 1 with 230 and set 2 with 262 mRNAs) were finally selected for diagnosis and prognostic miRNA targets. The miRNA-mRNAs interactions were further visualized for better understanding using Cytoscape3.7 (Shannon et al., 2003).

### Pathway enrichment analysis

The previously identified genes (230) of set and 262 were further used for the identification of pathways and other molecular functions, network and toxicity enrichment analysis in the IPA (Ingenuity Pathway analysis). The IPA can analyze enriched biological pathways, disease and functions, upstream and downstream interactions, molecular function and toxicity for a given gene set. It also gives the statistical significance for each enriched process.

### Gene ontology (GO) enrichment

The Gene Ontology is a vocabulary developed to compare and identify genes based on the processes in which they are involved. The GO is defined into three categories viz. Biological Process (BP), Molecular Function (MF), and Cellular Component (CC). In the current studies, the enrichment analysis was performed using PANTHER (Mi et al., 2019) (http://www.pantherdb.org/geneListAnalysis.do) and enrichR (https://maayanlab.cloud/Enrichr/) with *p*-value < 0.001 for identification of significant GO terms.

## Results

One of the goals of this study was to develop an efficient miRNA signature to correctly classify the early and late stage patients using miRNAs expression profiles using the machine learning algorithms. Further, a least absolute shrinkage and selection operator (LASSO) penalized regression model was employed to identify signature miRNAs for their prognostic potential. The network and pathways analysis revealed that several target genes involving various tumor suppressor and oncogenes in cancer regulatory pathways may be affected by the aberrant expression of the identified miRNAs. The important results are given below.

### Differentially expressed microRNAs (DE-miRNAs)

**Figure 2:**
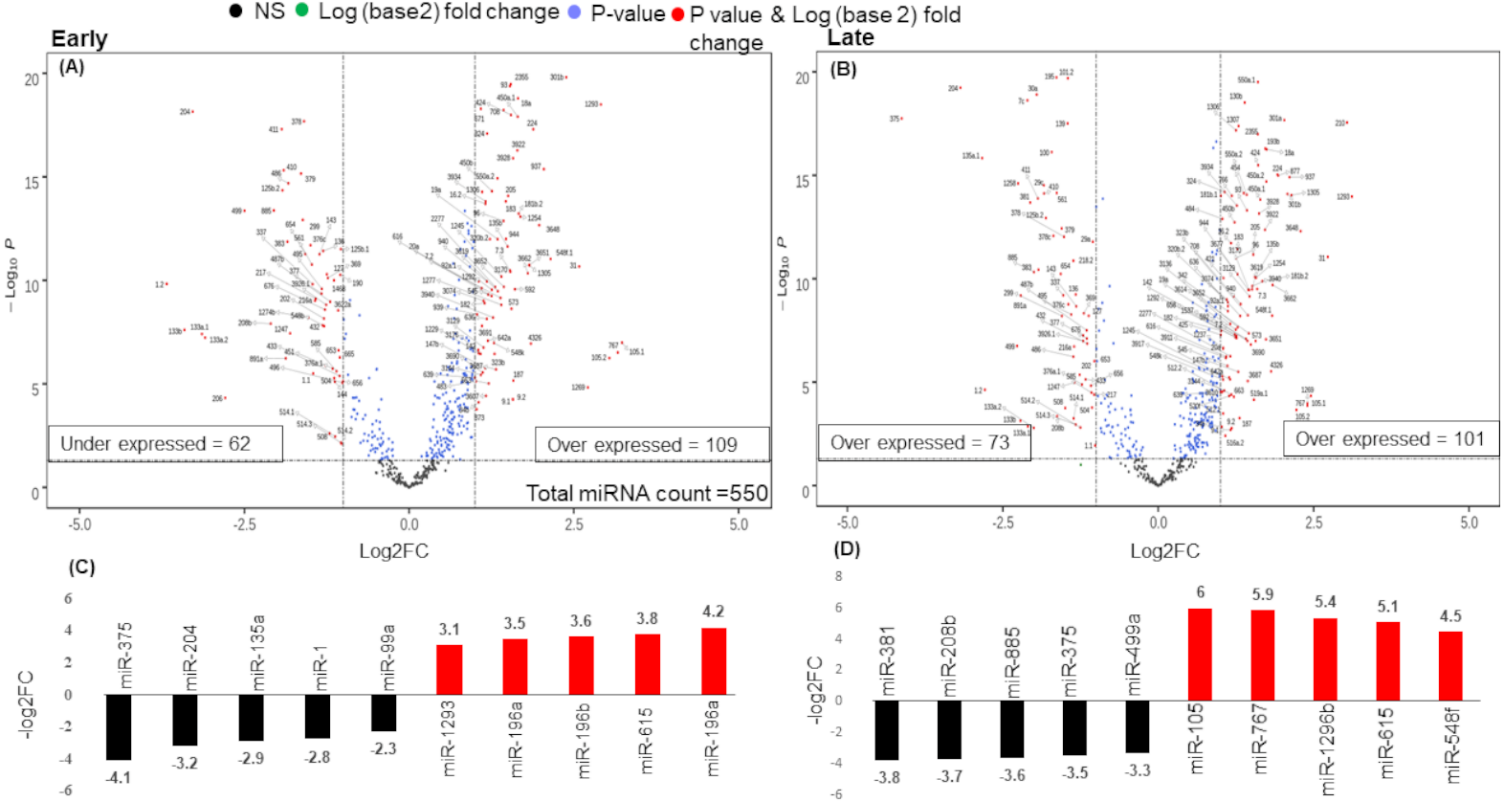
Volcano plot for expressed genes in (A) early stage (B) late stage. The miRNAs were considered significantly differentially expressed (DE-miRNA) if (|log2FC ≥ 1| and *p*-value < 0.05). Total 171 DE-miRNAs (under-expressed = 62 and over-expressed = 109) were identified in early stage whereas 174 DE-miRNAs (under-expressed = 73 and over-expressed = 101) were identified in the late stage. (C) Top five up (red) and down (black) regulated miRNAs in early stage (D) Top five up (red) and down (black) regulated miRNAs in late stage.

There were 171 miRNAs differentially expressed (62 over and 109 under-expressed) in early and 174 miRNAs differentially expressed (73 over and 101 under-expressed) in late stages (Figs. 2A & 2B) (Tables S2A & S2B). The top 5 over and under-expressed miRNAs are given in Figs. 2C and 2D. The heat map of differentially expressed miRNAs in early stage shows a visual distinction between the expression of miRNAs in normal and cancer tissues (Figs. S2A & S3B).

**Figure 3:**
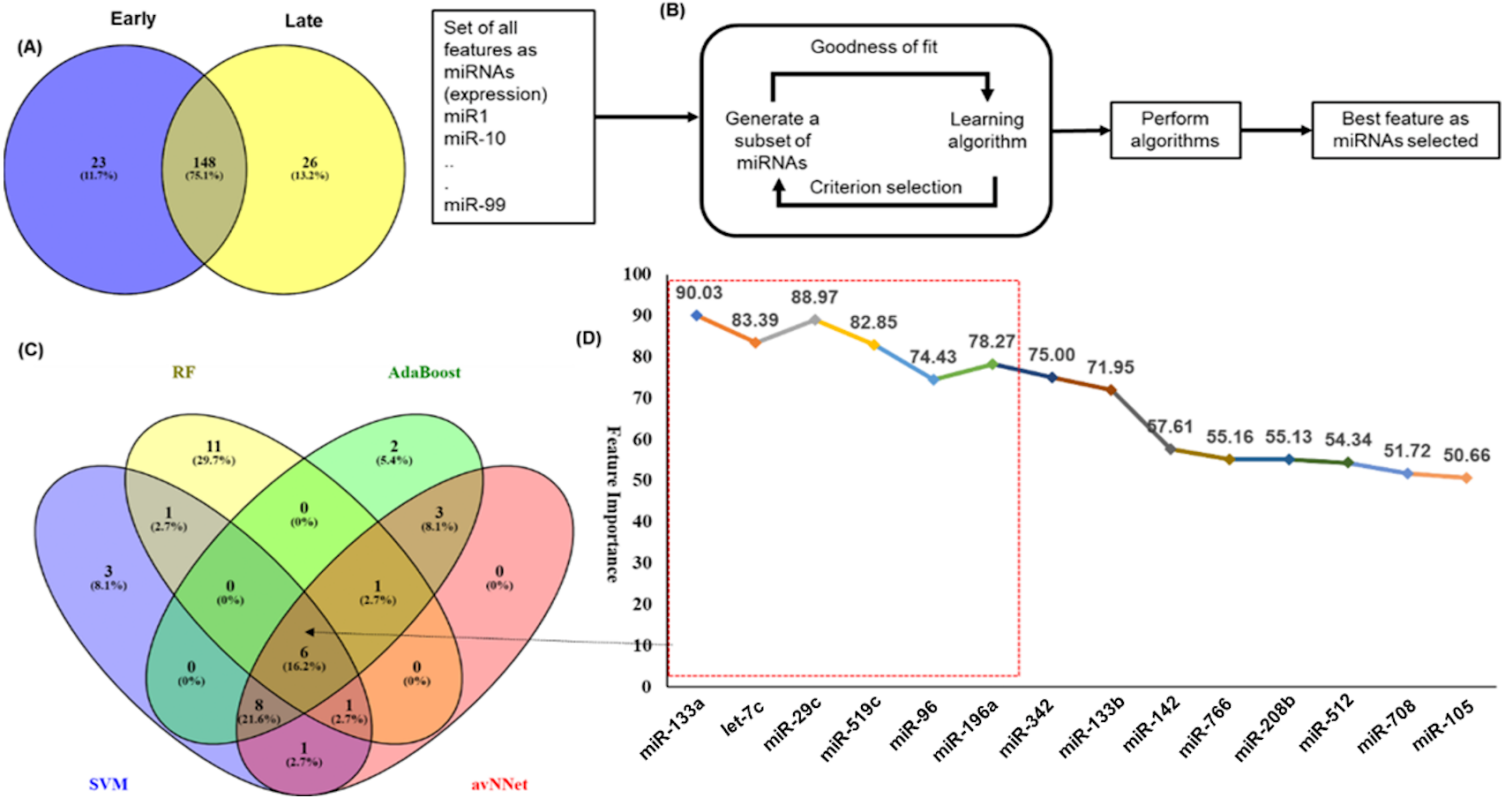
Feature selection for model building. The distribution of miRNAs into early and late stages. There were 23 miRNAs specific to early-stage while 26 miRNAs were specific to the late-stage. A total of 148 miRNAs were common to both the stages. (B) The schematic presentation of the feature selection in machine learning. (C) The common and specific features among top 20 features predicted by each of the five ML techniques. (D) The six common miRNAs (highlighted in red color box) predicted by all five ML methods and eight common in any three methods with the average importance for predictors.

An analysis of the common and stage specific miRNAs revealed that there are 148 miRNAs common to early and late stages, whereas 23 and 26 miRNAs are specific to early and late stages respectively (Fig. 3A) (Table S3). Similar to the DE-miRNAs analysis, the DE-mRNAs of 101 early stage with 44 normal adjacent tissues (NAT) samples were resulted in the 3831 differentially expressed mRNAs (2407 over and 1424 under-expressed) with (|log2FC ≥ 1| and *p*-value < 0.05) (Fig. S3).

### Identification of miRNA signature for classification of early and late stages

An important objective of this study was to identify the miRNAs signature for the early and late (TNM stage) using the miRNAs expression profiles. The signature miRNAs were identified using the ensemble machine learning feature selection method (Random Forest (RF), Support Vector Machine Radial Kernel (svmR), Adaptive Boost (AdaBoost), averaged Neural Network (avNNet), and Gradient Boosting Machine (GBM)). A detailed method for feature selection by minimum Redundancy Maximum Relevance (mRMR) algorithm is given in Fig. 3B. The top 20 predictor miRNAs were identified using each of the machine learning methods (RF, svmR, AdaBoost, avNNet and GBM) and the common among them were chosen for further model building and evaluation (Fig. 3C, Table S4). The ensemble ML based mRMR feature selection method reduce the bias that might be introduced by one or two methods. As a result, a 6 miRNA (let-7c, miR29c, miR-96, miR-133a, miR-196a, miR-519a) with average importance ranging from 90.03 to 78.27 for classification were selected for further studies. The mean importance of these miRNAs is shown in Fig. 3D. Among them, the miR-96 and miR-196a are over-expressed while the rest (miR-133b, miR-29c, miR-519a and let-7c) are under-expressed in normal vs primary tumor samples (Fig. S4). It is interesting to note that miR-519a is significantly differentially expressed in early stage only.

### The performance of generated models for stage classification

**Figure 4:**
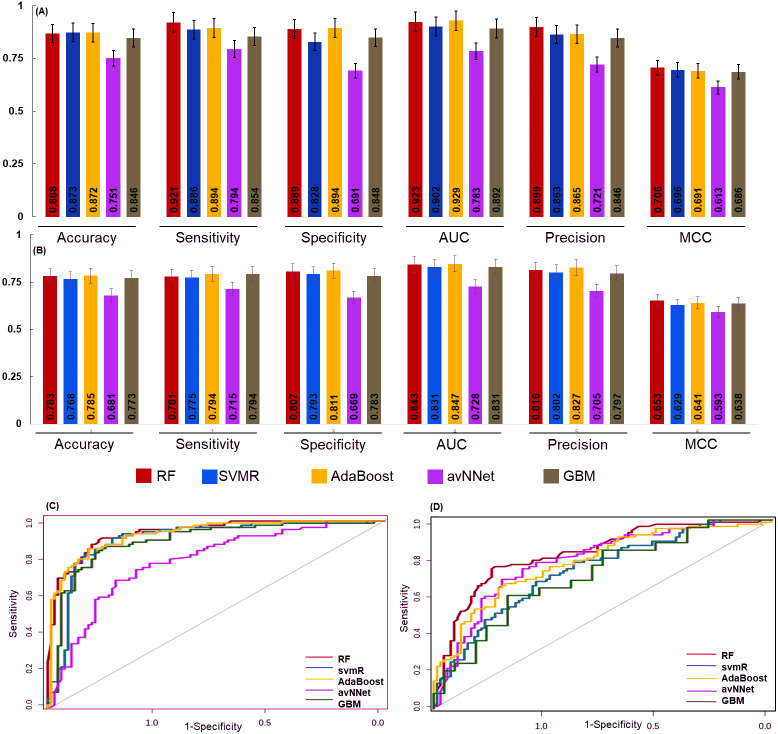
The performance of the generated model. The performance of the models was examined for accuracy, specificity, sensitivity and ROC generated for (A) over sampled and (B) under sampled data set. The model robustness was further evaluated by external data (test set) and AUC was shown in (C) oversampling and (D) under sampling. In both the sampling methods the generated models showed good classification accuracy. The estimated error bars shown as standard error.

The 6 miRNAs were selected by all five machine learning methods. The model was built using a training set and an independent test set (70:30) ratio was used for model prediction. The Synthetic Minority Oversampling Technique (SMOTE) algorithm was used for data balancing. In the under sampling total 101 (early and late stage) samples were used for internal model building and performance was evaluated using accuracy, sensitivity specificity, area under the curve (AUC), precision call and MCC of RF, svmR, AdaBoost, avNNet, and GBM. The accuracy of these five model was found to be 0.79 ± 0.03, 0.71 ± 0.04, 0.76 ± 0.03, 0.76 ± 0.03 0.79 ± 0.03, 0.71 ± 0.04, 0.76 ± 0.03, 0.76 ± 0.03, and 0.64 ± 0.042 for under-sampling methods. Similarly, the accuracy for RF, svmR, AdaBoost, avNNet and GBM was 0.87 ± 0.021, 0.86 ± 0.032, 0.87 ± 0.035, 0.85 ± 0.032, and 0.80 ± 0.038 respectively for oversampled data (early and late = 352 samples) The details of the internal top 10 models of ML is given in the Table S5. The detailed performance of each ML algorithm is given in Figs. 4A & 4B.

The final model performance was evaluated by the independent test set using accuracy, sensitivity, specificity, AUC, precision, and MCC. The accuracy for RF, svmR, AdaBoost, avNNet, GBM was found 0.87, 0.87, 0.87, 0.75, and 0.84 for test set generated using oversampled data. Whereas the accuracy was 0.79, 0.76 0.78, 0.68, 0.77 for the above five methods for the test set generated using under-sampled data . The MCC (0.65, 0.62, 0.64, 0.59, and 0.68 was for RF, svmR, AdaBoost, avNNet and GBM) gave better model performance over other confusion matrix categories. The robustness of model was evaluated using the area under curve (AUC) of Receiver Operating Characteristics (ROC) given in Figs. 4C & 4D for over and under sampled data. The RF and AdaBoost found the subsequently higher prediction accuracy in the under and over sampling methods and avNNet was the least prediction accuracy. The overall classification result showed, the generated 6 miRNAs signature can stratify the HNSCC patients on clinical TNM stages with reasonable accuracy based on expression profile.

### Risk score calculation

**Figure 5:**
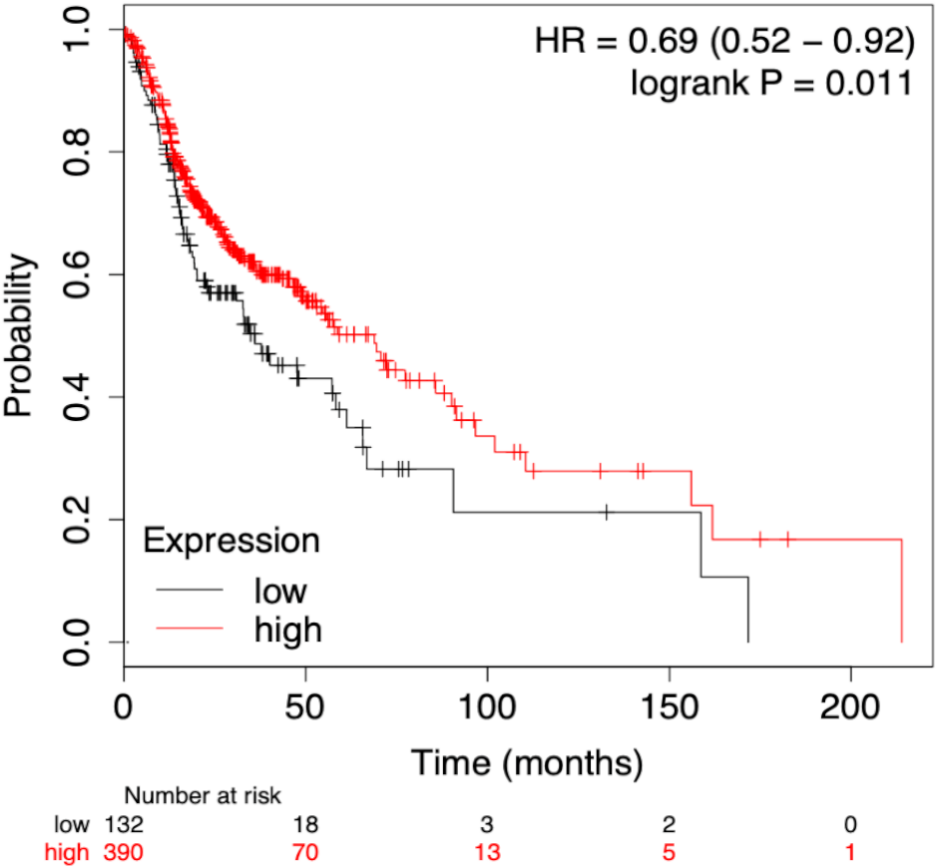
Survival plot of prognostic signature miRNA as biomarker. The cumulative effect of identified miRNA (eight in total) expression on the patient’s survival. The cox-proportional hazard analysis revealed that the miRNAs were significantly associated with the overall survival of the patients (*p*-value < 0.05).

To assess the prognostic performance of the generated model the time-dependent ROC curve was considered for risk score (low and high) calculation. The overall 5-year risk score was calculated of the normalized expression with the coefficient of the LASSO cox-coefficient. As stated in methodology, the risk score can be used to assess the risk for a patient based on the median value expression. The risk score was calculated using the risk score equation as given below and plotted using Kaplan-Meir plot Fig. 5. Kaplan-Meir plot for each of the signature miRNAs is given in Fig. S5. Risk Score = (0.022 × let − 7_expression_) + (−1.996 × miR-96_expression_) + (6.593 × miR-9_expression_) + (1.361 × miR-143_expression_) + (−2.938 × miR-379_expression_) + (−1.249 × miR-545_expression_) + (−1.969 × miR-658_expression_) + (−1.446 × miR-3926_expression_) − 1.505.

**Figure 6:**
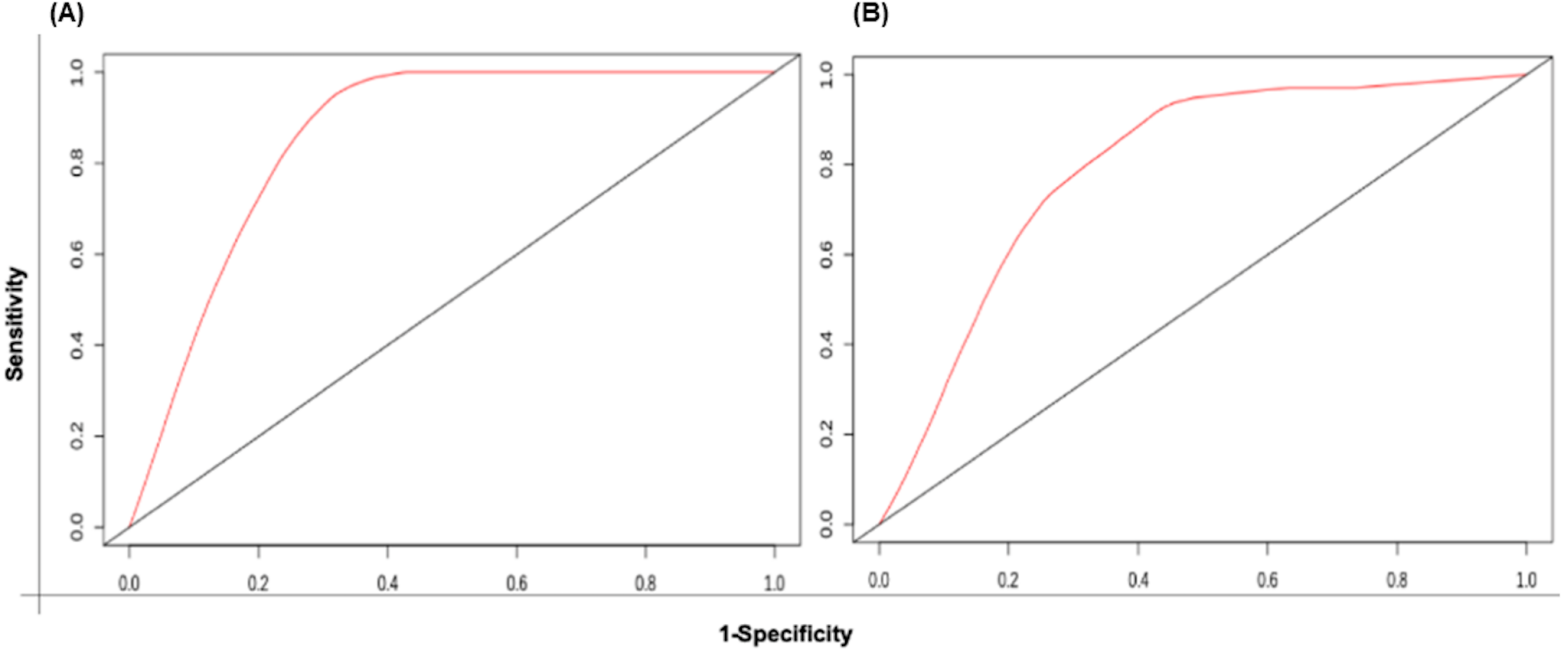
The time-dependent ROC curve for evaluation of the prognostic performance of the generated miRNA signature for (A) training set and (B) test set. The 5 years risk score prediction showed AUC of 0.86 in training set and 0.79 in the test.

The patient samples were systematically divided into training (368 samples) and test sets (160 samples). The 5 risk score (high and low) prediction was evaluated by AUC. The AUC for the training set and test set was found to be ∼0.86 and ∼0.79, respectively which indicated good performance of the identified signature (Figs. 6A & 6B). This time-dependent risk prediction analysis clearly showed that these miRNAs can be used for risk assessment of HNSCC patients.

### Univariate and multivariate analysis for prognostic evaluation

The identified miRNA signature was evaluated against various clinical variables to check their effect on survival using the standard Cox-proportional hazards regression (Table 1). It indicates that advancing pathological stages may have a significant adverse impact on the survival of the patients. The Cox univariate analysis showed that pathological stage, T stage, N stage, metastasis and signature miRNAs were significantly associated with the prognosis. While in multivariate analysis pathological T stage (*p*-value = 0.017) and signature mRNA (*p*-value = 0.023) showed significant *p*-values associated with the poor prognosis (Table S6).

### The microRNA-mRNA interactions

It was imperative to understand the role of the identified miRNA signatures in different biological processes. A mapping of the mRNA targets of the identified signatures (6 miRNAs for stage classification and 8 for prognosis) resulted in the identification of 1532 and 1446 mRNAs, respectively. Further, the analysis was done to select those mRNAs that get differentially expressed in various stages of HNSCC. It was found that for the identified diagnostic and prognostic miRNA signatures, there are 2 set of mRNA’ target (set 1 with 230 and set 2 with 262) mRNA that were differentially expressed respectively in the HNSCC patients (Tables S7 & S8).

**Table 1:** The univariate and multivariate Cox regression analysis revealed independent risk factors.

Variables	**Cox univariate analysis**		**Multivariate analysis**	
	*p*-value	HR (95% CI)	*p*-value	HR (95% CI)
Age (high vs. low)	0.54	1.3 (0.99–3.18)		
Pathological stage (I + II vs. III + IV)	0.0082*	1.7 (1.1–2.40)		
Pathological T stage (T1 + T2 vs. T3 + T4)	3.1e−05*	2 (1.5–2.8)	0.0175*	3.4 (1.24–9.42)
Pathological N stage (N0 vs. N1 + N2)	0.00075*	1.8 (1.3–2.6)		
Pathological metastasis (M0 vs. M1)	0.0021*	28 (3.3–230)		
Gender (Male vs. Female)	0.35	0.72 (0.54–0.98)		
Signature miRNAs risk (Low vs. High)	0.044*	1.4 (1–2)	0.0235*	1.68 (0.88–3.20)

### Biological pathways, diseases functions and network enrichment analysis

As indicated earlier, the pathways enrichment analysis was performed by the Ingenuity Pathway Analysis (IPA) separately for diagnostic targets (set 1) and prognostic targets (set 2).

**Figure 7:**
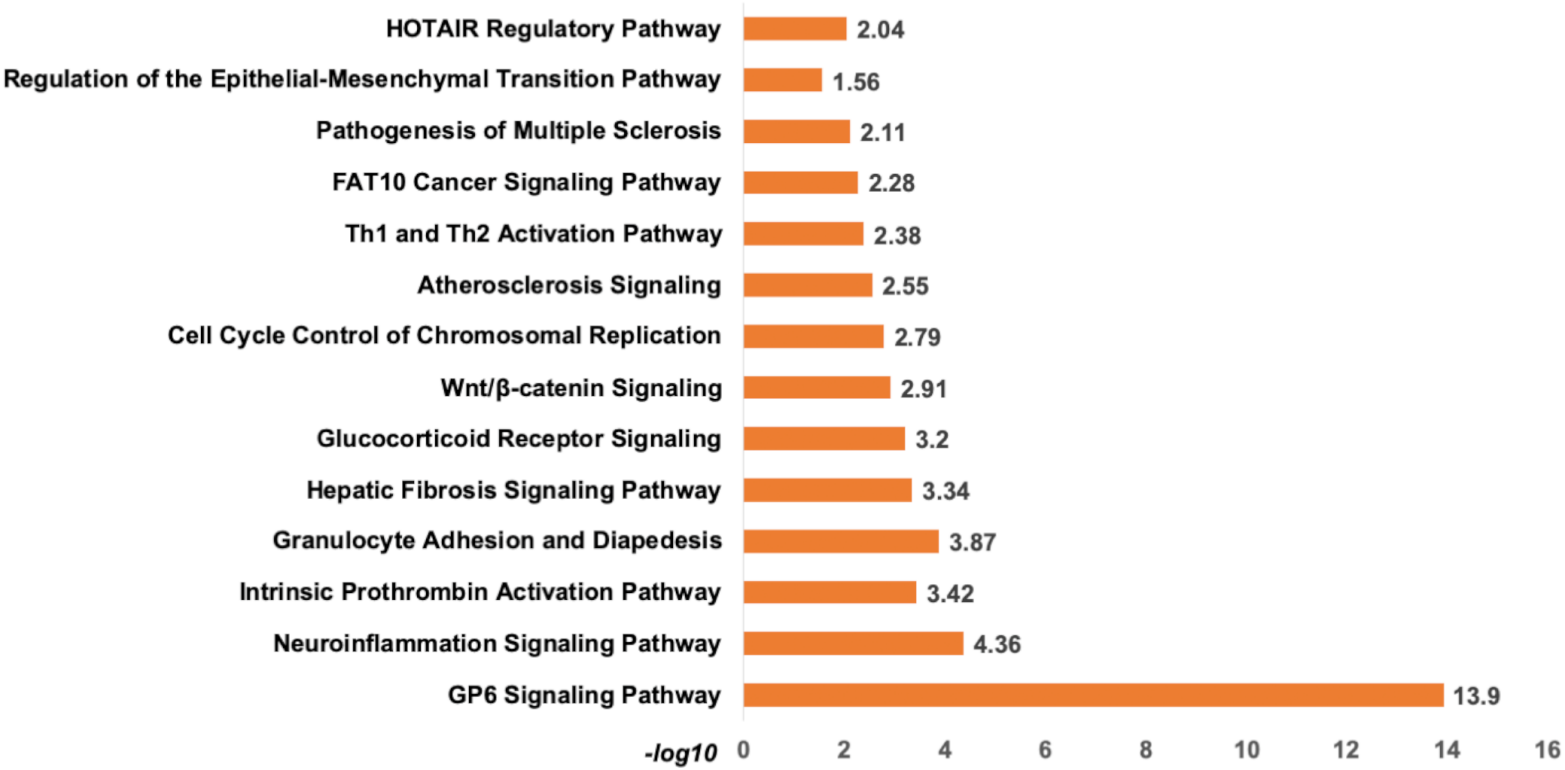
The pathway enrichment analysis of signature miRNA. The pathway enrichment analysis for the identified signatures of set 1 genes (230 mRNA). The top most enriched pathways of diagnostic signature miRNA targets genes in IPA.

**Figure 8:**
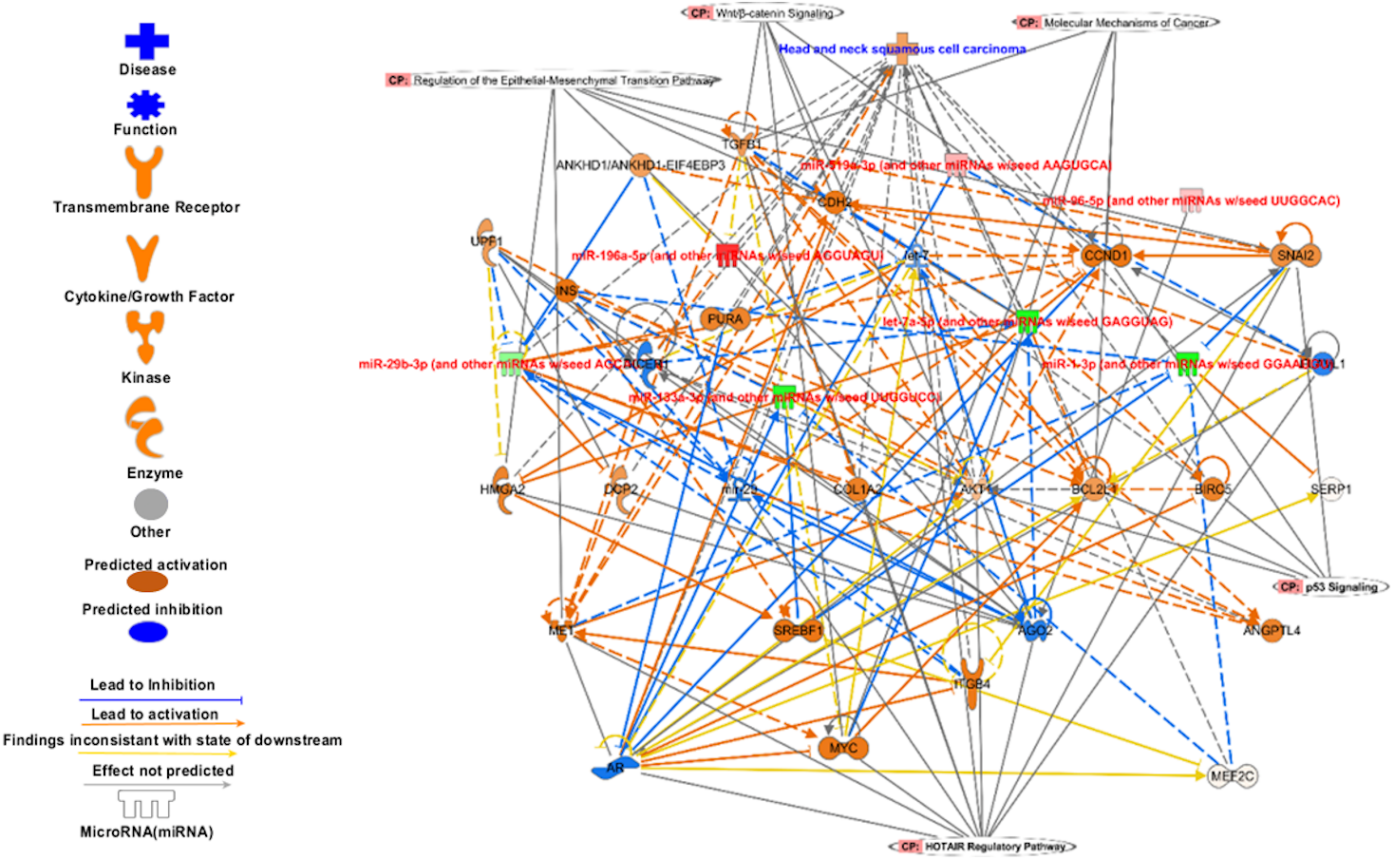
The signature miRNAs targets different pathways genes. The targets of signature miRNA in different pathways. The signature miRNA are regulating key cancer pathways e.g., Epithelial-Mesenchymal Transition, Wnt/*β*-catenin signalling, molecular mechanism of cancer, p53 signalling, and HOTAIR regulatory pathways.

*Analysis of set 1:* The top enriched pathways for diagnostic targets were (i) GP6 signalling (ii) Neuroinflammation signalling (iii) Intrinsic Prothrombin Activation Pathway (iv) granulocyte adhesion and diapedisis (Fig. 7). Other enriched pathways are given in Table S9. The KEGG pathways analysis also revealed that majority of the genes were associated with pathways such as cytokine-cytokine receptor interaction, small cell lung cancer, transcriptional misregulation in cancer, ECM-receptor, and glutathione metabolism (Table S10). While WikiPathways enrichment showed, nuclear receptors meta-pathways (ID: WP2882), TGF-beta signalling pathways (WP366), PI3K-Akt-mTOR-signalling pathway (WP4172) (Table S11). The pathway analysis revealed that these miRNAs targets are highly enriched with cancer associated pathways. The top disease and disorders associated with the set 1 are organismal injury and abnormalities, cancer, connective tissue disorder, and skeletal muscle disorder. Top molecular functions and cellular functions were linked with cellular movement, cellular assembly and organization, cellular function and maintenance, cell death & survival and cell development (Fig. 8) (Table S12). The top upstream regulators were miR-29c, TGFB1, estrogen receptor, histone h4, and CREB which are associated with cell growth and proliferation. The miR-29, a potent tumor suppressor, is found under-expressed in this study. Interestingly, the top analysis of the upstream regulatory genes showed that miR-29 directly targets 11 genes (9 over-expressed and 2 under-expressed) associated with head and neck cancer (Fig. S6). A thorough look at these mRNAs revealed that many of them are transcription factors (total 31) and part of cancer gene panels (total 19) (Sondka et al., 2018). These mRNAs were used in the subsequent functional analysis step. It was imperative to note that many of the transcription factors and cancer censes gene are targeted by the let-7c which is well known for its role in a variety of cancers (Fig. S7). The toxicity enrichment analysis revealed increased levels of alkaline phosphatase (ALP). Interestingly, the serum ALP level have been found to be significantly higher in OSCC patients (Acharya et al., 2017).

**Figure 9:**
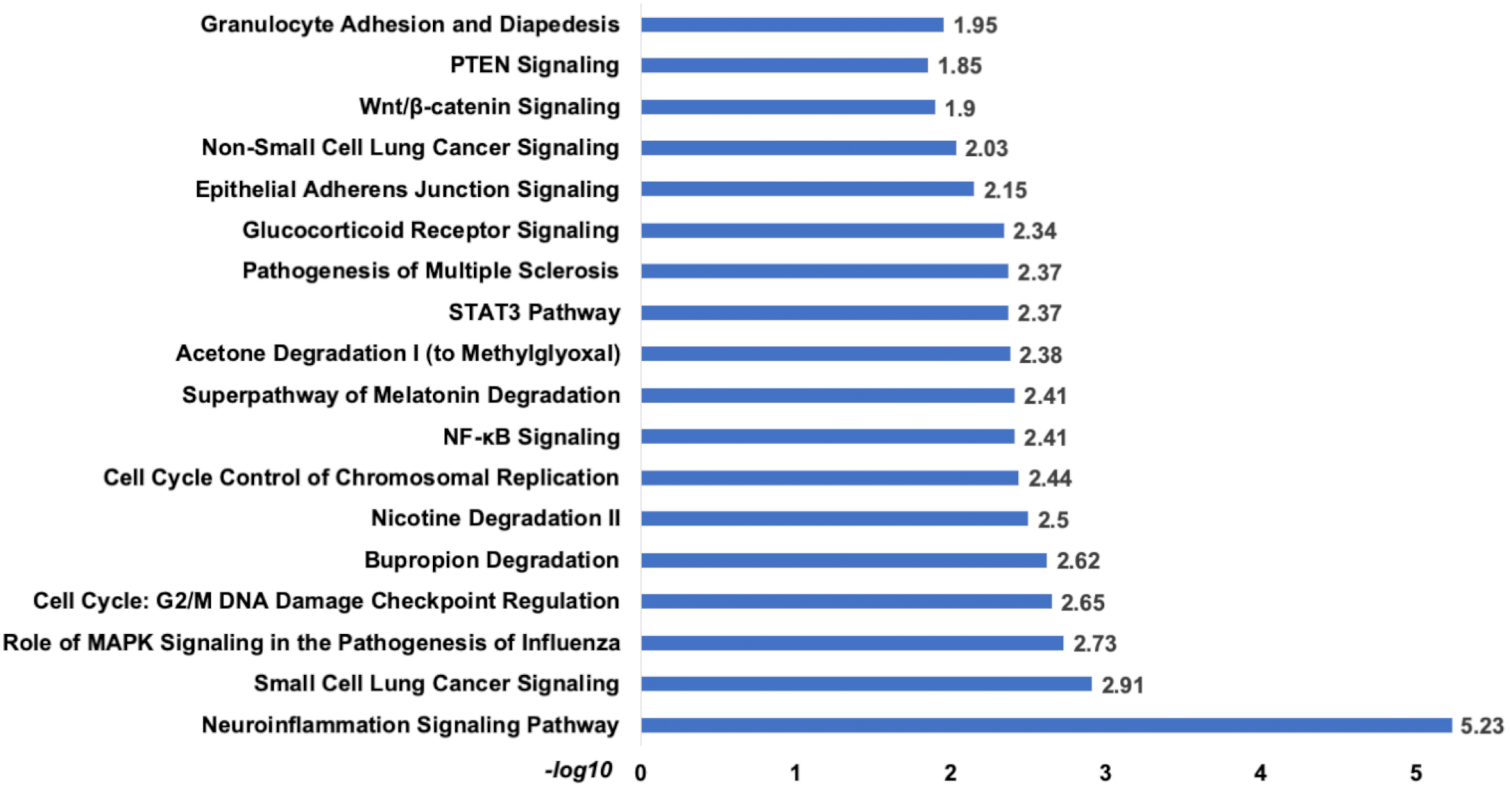
The pathways enrichment analysis for the identified signatures of set 2 genes as prognostic marker. The enriched biological pathways by IPA. The prognostics signature miRNAs are mostly enriched cancer-related pathways such as small cell lung cancer, Cell-Cycle:G2/M DNA damage checkpoint regulation, NF-kappa B signalling, etc.

**Figure 10:**
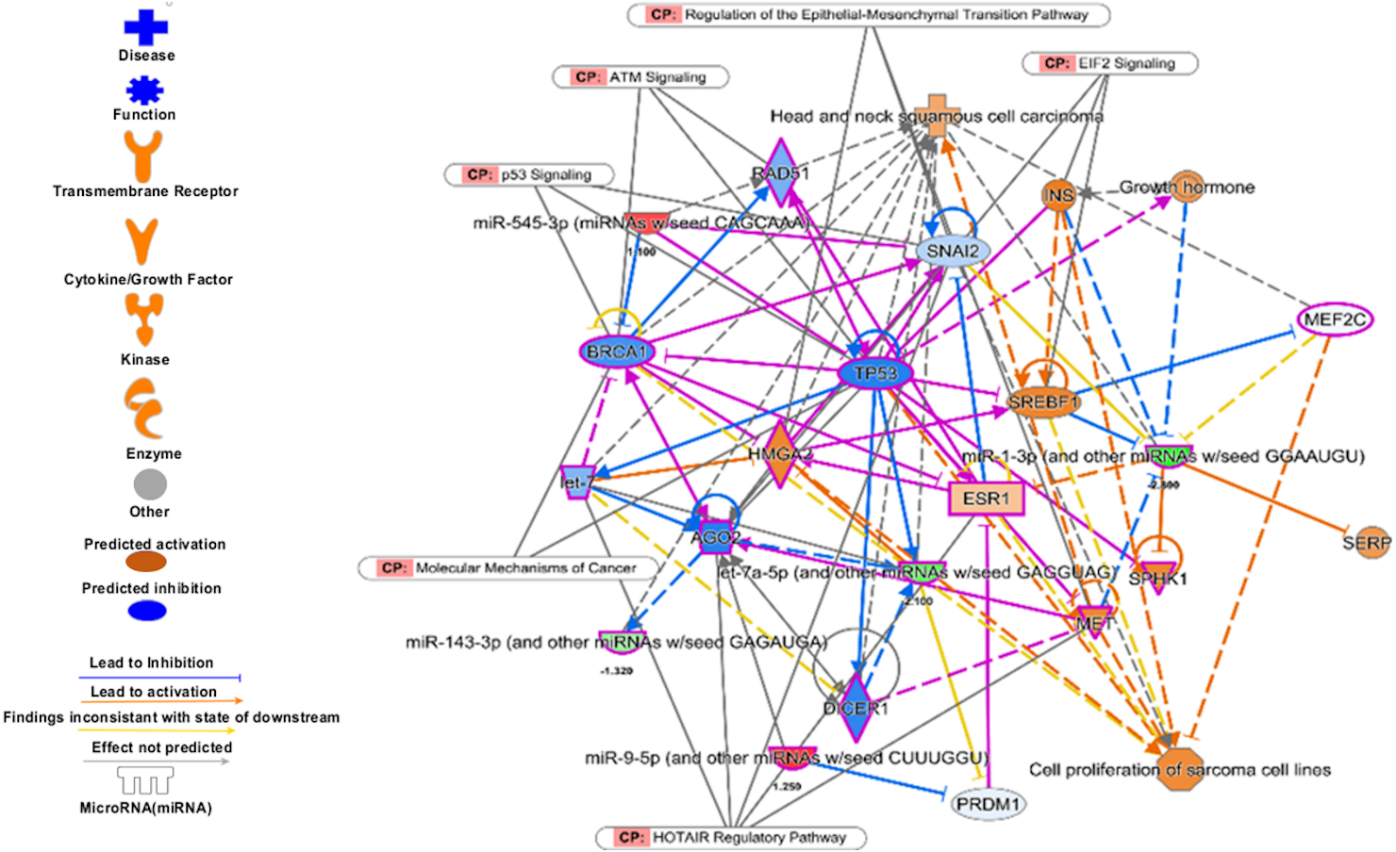
The prognostics signature target different carcinogenic gene and pathways. The molecular target of these eight signature miRNA were predominantly associated with tumor-suppressors and oncogenes in different pathways. Most of the miRNA targets are directly associated with HNSCC progression and proliferation. These miRNAs also targeting different cancer regulatory pathways i.e., p53 signalling, ATM signalling, regulation of the EMT pathways, EIF2 signalling, HOTAIR Regulatory pathways, and molecular mechanisms of cancer. The pink marked mRNAs are known to be associated with head and neck cancer.

*Analysis of set 2:* The pathway enrichment analysis by IPA showed that the top enriched pathways are neuroinflammation signalling, small cell lung cancer, MAPK signalling, and cell cycle: G2/M DNA damage checkpoints (Fig. 9) (Table S13). Top prognostics signature miRNAs are directly targeting several key cancer regulatory pathways such as p53 signalling, ATM signalling, EMT pathways etc., (Fig. 10). The KEGG pathways analysis also revealed that majority of the genes were associated with cancer progression such as protein digestion and absorption, ECM-receptor, transcriptional misregulation in cancer, and focal adhesion. In case of WikiPathways, the ECM and membrane receptors (ID: WP291), miR-509-3p alteration of YAP1/ECM axis (WP3967), and focal adhesion-PI3K-Akt-mTOR-signalling pathway (WP3932) were found enriched (Table S14).

The top regulatory effect networks were MITF, CD3, YAP1, let7, TGF *β*1, and TNF. MITF is mitochondrial transcription factor genes and have essential roles in cell differentiation, proliferation and survival of cell (Bertolotto et al., 1998). It is also important co-activator of TGF *β* (Nijman et al., 2006). CD3 is cluster of differentiation 3 activates in early oral premalignant (Öhman et al., 2015). The Hippo-YAP1 pathway is oncogenic in head and neck cancer (Santos-de Frutos, Segrelles & Lorz, 2019). TGF *β*1 dysregulation is very common in several cancers including HNSCC. It is key regulator of epithelial cell proliferation, growth factor and angiogenesis (Rothenberg & Ellisen, 2012). The dysregulation of TNF genes are involved in almost all types of cancer. The TNF plays an important role in cytokine production and have critical role in immune regulation (Alsahafi et al., 2019).

The top diseases and disorders found to be associated with the genes of this set were cancer, organismal injury & abnormalities, and hematological disorders. Additionally, top associated network functions were associated with cell death and survival, cellular development, cellular growth, and proliferation (Table S15). The miRNA-mRNA interaction analysis showed that there are 16 mRNAs which are part of cosmic consensus cancer gene panel and there are 20 transcription factors among the targets. Six mRNAs were common to transcription factors and cancer gene panels (Fig. S8). Overall enrichment analysis revealed that these miRNAs were directly or indirectly associated with the tumorigenesis and poor prognosis.

### Gene ontology enrichment analysis

*Set 1:* The most enriched biological processes were associated with extracellular matrix proteins organization (GO:0030198), regulation of natural kill cell chemotaxis (GO:2000501) and response to alcohol (GO:0097305). While top molecular functions enriched genes were mRNA 3′-UTR binding (GO:0003730), chemokine activity (GO:0008009) and transforming growth factor beta-activated receptor activity (GO:0005024). The cellular component such as messenger ribonucleoprotein complex (GO:1990124), serine/threonine protein kinase complex (GO:1902554), gamma-tubulin large complex (GO:0000931) were found majority of genes involved in these process (Fig. S9).

*Set 2:* Many of the enriched GO processes are similar to set 1. However, there are notable differences as well. The most enriched genes were involved in biological process such as collagen fibril organization (GO:0030199), negative regulation of myeloid cell differentiation (GO:0045638), and negative regulation of cell differentiation (GO:0045596). In the molecular functions enriched genes were platelet-derived growth factor binding (GO:0048407), transcriptional activator activity, RNA polymerase II transcription regulatory region sequence-specific binding (GO:0001228), cell proliferation (GO:0008283), metabolic process (GO:0008152), biological adhesion (GO:0022610). The endoplasmic reticulum lumen (GO:0005788), integral component of plasma membrane (GO:0005887), and intermediate filament (GO:0005882) were most enriched cellular component terms (Fig. S10).

## Discussion

The HNSCC is associated with high mortality and morbidity. It is highly heterogeneous and identification of robust, potential and reproducible biomarker is a major challenge. The TCGA provides detailed transcriptomic data with clinical information that can be gainfully used for better risk prediction and disease management. The ML techniques, riding the recent advancement in software, hardware, and sequencing technologies, are being used to identify patterns or factors (mutation/variation) for personalized diagnostics, prognostics, and therapeutics (Kann et al., 2019; Topol, 2019). The alteration in miRNA expression in tissue as well as in biological fluids has been reported to be an important biomarker in various cancers. Interestingly, during the course of this work, we came across the announcement by Toshiba company (https://www.toshiba.co.jp/rdc/rd/detail_e/e1911_06.html) about a device that can predict the presence of 13 different cancers using the miRNA expression in blood alone with high (>99%) accuracy. Thus proving beyond doubt that miRNA expression data can be utilized for the development of a simple yet highly accurate screening tool for various cancers.

The etiology and aggressive behavior of HNSCC are not well understood at the molecular level. It calls for the studies to improve the molecular understanding leading to overall better clinical outcomes. Therefore, the main purpose of this study was to identify a set of signature miRNA using ML techniques to correctly classify the early and late-stage along with other prognostic biomarkers. The main findings of the study are outlined below:

*The machine learning techniques identified biomarkers with good accuracy:* The analysis resulted in the identification of 6 miRNA signature which predicted the early and late stages with good accuracy on external data set. The good accuracy in classification of the samples in the external set further validated the good classification ability of the generated models. It is pertinent to note that the stage is also a significant prognostic indicator.

The evaluation of the identified prognostic signature indicated that these miRNAs are significantly associated with poor survival of the patient and thus it can be a good prognostics biomarker. The IPA, KEGG and WiKipathways analysis also revealed that target of these miRNAs are highly associated with the cancer initiation and progression. The molecular functional analysis of the identified miRNA also indicated that they directly or indirectly regulate several oncogenes, tumor suppressor genes and transcription factors.

*The data balancing by enhanced sampling can improve accuracy:* The small cohort and imbalanced data highlight the major challenges in this study. The SMOTE algorithm was therefore utilized to generate a balanced dataset for classification of stages (Vafaee et al., 2018). It was interesting to note that the models generated with oversampling of the data showed better accuracy as compared to the undersampled data. Perhaps, the undersampling of the data might have resulted in loss of important information and thus reduced accuracy.

*The identified miRNAs are detectable in biological fluids:* A detailed literature survey suggested that the identified miRNAs are related to diagnostics and prognostics of many cancers. Interestingly, they are also found in biological fluids (plasma, serum, etc.) in several cancers including HNSCC. The miR-29 and let-7 family miRNAs were significantly downregulated (*p* < 0.001) in the serum of patients with high-risk oral lesions. The miR-196a was found up-regulated in the plasma of HNSCC patients. The miR-486 was found downregulated in blood samples of non-small lung cancer patients. The reduced expression of circulating miRNA-133b was also associated with the clinical stage, metastasis, and survival of breast cancer patients. The upregulation of miR-96 is associated with various cancers such as breast (Zhang et al., 2017), hepatic (Musaddaq et al., 2019), and colorectal (Brunet Vega et al., 2013). The deregulation of miR-519 is linked with the prognosis and diagnosis of lung cancer and it’s found to be in circulating in plasma (Wang et al., 2019). The overall literature survey suggested that the identified miRNAs were found dysregulated in biological fluids of patients of various cancers. They can also be useful for the detection and prognosis of HNSCC in biological fluids as well, though experimental validation would be needed to prove this assumption.

## Conclusion

In this study we have tried to identify miRNA expression patterns for classification of TNM stage (early and late stage) in HNSCC patients using machine learning algorithms (RF, svmR, adaBoost, avNNet and GBM). The dissection of miRNA expression with the help of machine learning tools provided us with miRNA signatures that can distinguish between early and late-stage tumor samples and risk to the patient with good accuracy. The five-year overall survival analysis revealed that the dysregulation of the identified miRNAs is significantly correlated with poor prognosis. We have further identified the functional roles of the identified miRNAs, their mRNA targets, pathways and process that may get perturbed in the HNSCC carcinogenesis and progression. Very interestingly, the identified miRNAs were also found to be differentially expressed in the biological fluids of patients of HNSCC patients. This opens a possibility that these miRNAs can also be proposed as non-invasive biomarkers for HNSCC, although it will need a large number of patient samples and experimental validation. We hope that these studies will help in the development of potential biomarkers and miRNA based therapies in cancer.

##  Supplemental Information

10.7717/peerj.9656/supp-1 Table S1 The details of clinical information of Head and Neck Squamous Cell Carcinoma of individual patients from the TCGA NA—Not available

10.7717/peerj.9656/supp-2 Table S2 List of differentially expressed miRNAs of early and late-stage The list of differentially expressed in of early-stage (|log2 ≥ 1| *p*-value < 0.05) in samples (*N* = 101) and normal sample = 44 and late-stage (*N* = 352). The total differentially expressed miRNAs are normal vs early *N* = 171 & late *N* = 174)

10.7717/peerj.9656/supp-3 Table S3 List of common and stage specific differential expressed miRNAs A total of 148 miRNAs were common differentially expressed in early and late. In early 23 and 26 late-stage specific differential expressed miRNAs.

10.7717/peerj.9656/supp-4 Table S4 The top 20 important features identified by each machine learning algorithm (svmRadial, avNNet, Adaboost and RF) List of top 20 miRNA as an important feature variable for classification of early and late stages based on an expression. The best feature prediction showed AdaptiveBoost, Random Forest, Support Vector Machine and Average Neural Network.

10.7717/peerj.9656/supp-5 Table S5 Details of internal cross validation for diagnostic signature (ROC, Sensitivity, Specificity, Accuracy) over and under sampling The details of the model performance of individual algorithms i.e., SVM, RF, AdaBoost, avNNet, and GBM.

10.7717/peerj.9656/supp-6 Table S6 LASSO-Cox proportional hazard regression analysis for prognostic marker identification The LASSO based Cox proportional hazard regression was performed for the assessment of the prognostic value of patients. The star represent the significance of the p-value stastistics (* = 0.05, ** = 0.005 and *** = 0.0005).

10.7717/peerj.9656/supp-7 Table S7 The common target of the diagnostic signature miRNAs and differential expressed mRNAs (set 1) Details of the miRNAs target mRNAs and differential expressed log^2^FC.

10.7717/peerj.9656/supp-8 Table S8 The common target of the prognostic signature miRNAs and differential expressed mRNAs (set 2) Details of the miRNAs target mRNAs and differential expressed log^2^FC.

10.7717/peerj.9656/supp-9 Table S9 List of enriched pathways for diagnostic signature miRNA (set 1) The pathway analysis was performed using Ingenuity pathway analysis. The pathway enrichment was assessed by their enrichment −log_10_ *p*-value.

10.7717/peerj.9656/supp-10 Table S10 Pathways enrichment analysis of set 2 genes in KEGG (*p*-value < 0.05) The miRNA targets mRNAs were also enriched with the Kyoto Encyclopedia of Genes and Genomes (KEGG). The enriched pathways as evaluated based on their respective *p*-value.

10.7717/peerj.9656/supp-11 Table S11 Pathways enrichment against WikiPathways of set 1 genes (*p*-value < 0.05) The pathway analysis was performed using WikiPathways data set. WikiPathways contains the experimental biological data from different sources. The significant pathway is evaluated based on the *p*-value. The top enriched pathways are Nuclear Receptors Meta-Pathway, TGF-beta pathways, and PIK3-Akt signaling pathway.

10.7717/peerj.9656/supp-12 Table S12 IPA disease enrichment analysis for set 1 genes IPA has multiple tools for enrichment factor analysis such as disease and network. The disease enrichment analysis showed revealed cancer, Cell cycle and cell movement have a higher score (42,35,35 respectively).

10.7717/peerj.9656/supp-13 Table S13 IPA enrichment analysis for set 2 genes (Prognostic marker) The pathway analysis showed that top enriched pathways are Neuroinflammation Signaling (−log_10_ *p*-value = 5.23), Cell Cycle: G2/M DNA Damage Checkpoint Regulation (−log_10_ *p*-value = 2.65), Bupropion Degradation (−log_10_ *p*-value = 2.62). The complete list attached.

10.7717/peerj.9656/supp-14 Table S14 Pathways enrichment analysis of set 2 genes in KEGG (*p*-value < 0.05) The KEGG (Kyoto Encyclopedia of Genes and Genomes) is a systematic knowledge-based database repository of genes. Genes were systematically enriched into KEGG and found most of the pathways are associated with cancer progression such as ECM-receptor interaction signaling, small cell lung cancer, transcriptional misregulation in cancer has the highest significant *p*-value.

10.7717/peerj.9656/supp-15 Table S15 IPA enrichment network for set 2 genes IPA has multiple functional enrichment analysis suits. The top disease enrichment are mostly similar to the set 1 genes.

10.7717/peerj.9656/supp-16 Supplemental Information 16 The balancing of data set by SMOTE (Synthetic Minority Overampling TEchnique) algorithm The data set was balanced by under and oversampling methods using SMOTE package in R. In under-sampling, the majority class of data set was reduced (A) and in oversampling method, minority class of samples was amplified (B) to balance both datasets.

10.7717/peerj.9656/supp-17 Supplemental Information 17 Heat map for expression of differentially expressed miRNAs The early-stage miRNAs expression profile (normal adjacent (*N* = 44) vs tumour (*N* = 101)) (A). The late-stage miRNAs expression profile (normal adjacent (*N* = 44) vs tumour (*N* = 352)) (B). The heat map clearly shows the difference in expression profile between the normal and tumour samples. The heatmap was designed in R using complex heatmap library.

10.7717/peerj.9656/supp-18 Supplemental Information 18 The volcano plot for mRNA expression in the early stage of HNSCC The significantly differentially expressed (|log2fc ≥ 1|*p*-value ≤0.05) mRNAs are shown as red dots. A total of 3831 mRNAs were found to be differentially expressed (2407 over and 1424 underexpressed).

10.7717/peerj.9656/supp-19 Supplemental Information 19 Differential expressed signature miRNAs in early and late-stage Differential expression of signature miRNAs in early (A) and late-stage (B) (|log2FC ≥ 1| *p*-value ≤ 0.05). In early-stage (miR-196a, miR-96) are overexpressed in early and late while (miR-29c,let-7c and 133a-1) are underexpressed in both early and late stage. The miR-519a is only overexpressed in early stage.

10.7717/peerj.9656/supp-20 Supplemental Information 20 The Kaplan-Meier survival analysis for the identified prognostic signature (A–H) The low and high expression was classified based on the median value of the expression. The plots were generated from the KM plotter (*p*-value ≤ 0.05). The let-7c, miR-9, miR-96, miR-658, and miR-545 are positively poor survival correlated with overexpression of the patients while miR-143,miR-3926 and miR-379 are negatively correlated.

10.7717/peerj.9656/supp-21 Supplemental Information 21 The miRNA-mRNA interaction diagram The miR-29c is a potential biomarker under-expressed in HNSCC. There are nine of its target mRNAs (out of 11) were found to be over-expressed in HNSCC.

10.7717/peerj.9656/supp-22 Supplemental Information 22 The miRNA-mRNA interaction for the diagnostic signature The mRNAs in light pink circle are over-expressed whereas those in the light blue are under-expressed. The let-7c has a number of targets comprised of the transcription factors (shown in hexagonal shape, *N* = 31) and cancer catalogue genes (shown in triangle shape, *N* = 19). The genes which are both transcription factors and are cancer census genes are shown in diamond.

10.7717/peerj.9656/supp-23 Supplemental Information 23 The prognostic signature miRNAs and their mRNA targets The over-expressed mRNAs are represented in light pink and under-expressed are shown in light blue. The let-7c, miR-143, miR-3926, and miR-1 were found underexpresseed while majority of their mRNA targets are found over-expressed. Similarly, the miR-658, miR-9 and miR-545 were found up and their targets were found under-expressed. There are 20 transcription factors among the targets while 16 mRNAs are on cancer gene panels. Six mRNAs are common among the transcription factors and cancer census genes.

10.7717/peerj.9656/supp-24 Supplemental Information 24 The Gene ontology enrichment analysis revealed the enriched GO processes of set (1) The binding and catalytic activity were overrepresented in molecular functions class. In molecular functions, the binding and catalytic activity of the protein is most enriched while the translation of regulatory activity is least. The cellular process and metabolic process were the most enriched biological process, while the cell and organelle with extracellular region were the top mapped cellular process.

10.7717/peerj.9656/supp-25 Supplemental Information 25 The Gene ontology enrichment analysis in prognostic as set 2 The enriched GO process were found to be quite similar as of the diagnostics markers as described earlier.

## Additional Information and Declarations

### Data Availability

The following information was supplied regarding data availability: Raw data are available at TCGA under project ID TCGA-HNSC.
