# Supplementary figures and images for: Predictive models for stage and risk classification in head and neck squamous cell carcinoma (HNSCC)
Source: PeerJ. 2020 Sep 22;8:e9656. doi: 10.7717/peerj.9656 (PMC7518185; doi:10.7717/peerj.9656)

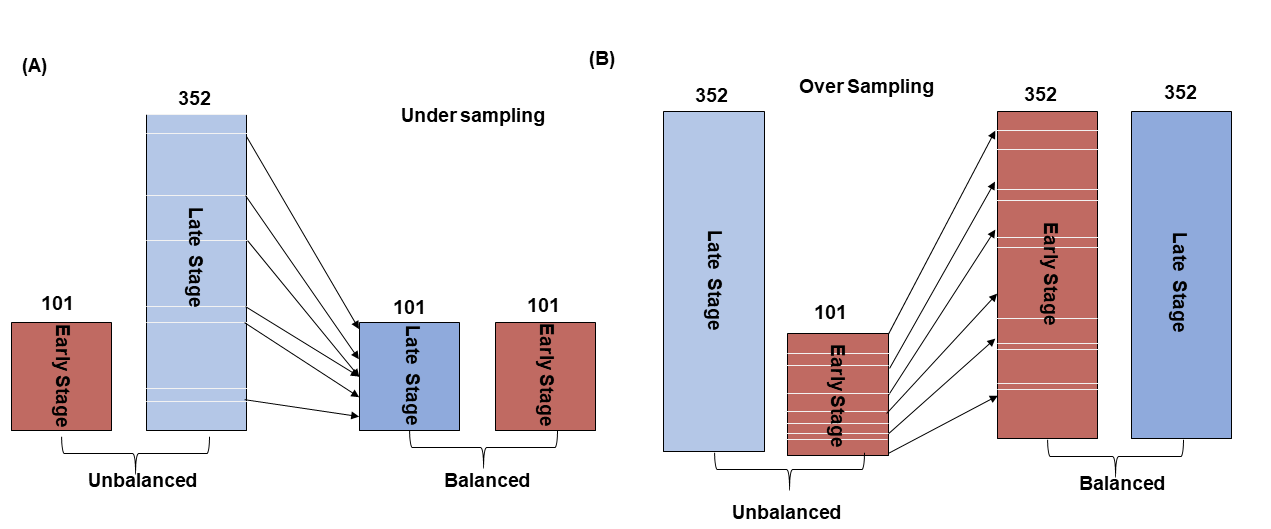

Supplement: Supplemental Information 16 — The data set was balanced by under and oversampling methods using SMOTE package in R. In under-sampling, the majority class of data set was reduced (A) and in oversampling method, minority class of samples was amplified (B) to balance both datasets. [file peerj-08-9656-s016.png]

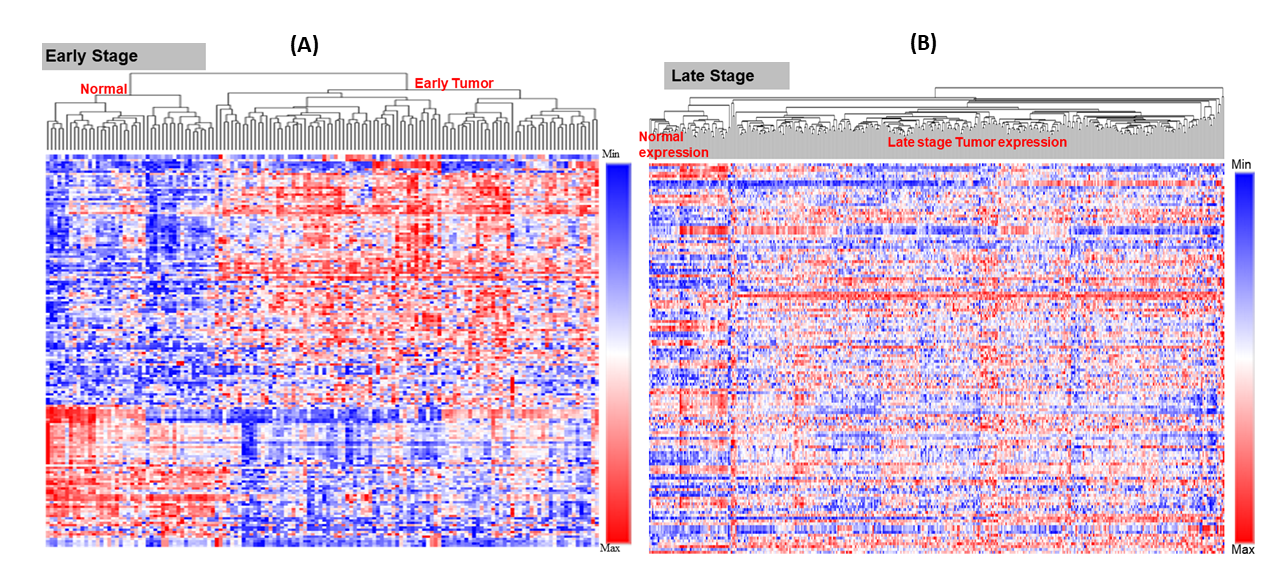

Supplement: Supplemental Information 17 — The early-stage miRNAs expression profile (normal adjacent (N = 44) vs tumour (N = 101)) (A). The late-stage miRNAs expression profile (normal adjacent (N = 44) vs tumour (N = 352)) (B). The heat map clearly shows the difference in expression profile between the normal and tumour samples. The heatmap was designed in R using complex heatmap library. [file peerj-08-9656-s017.png]

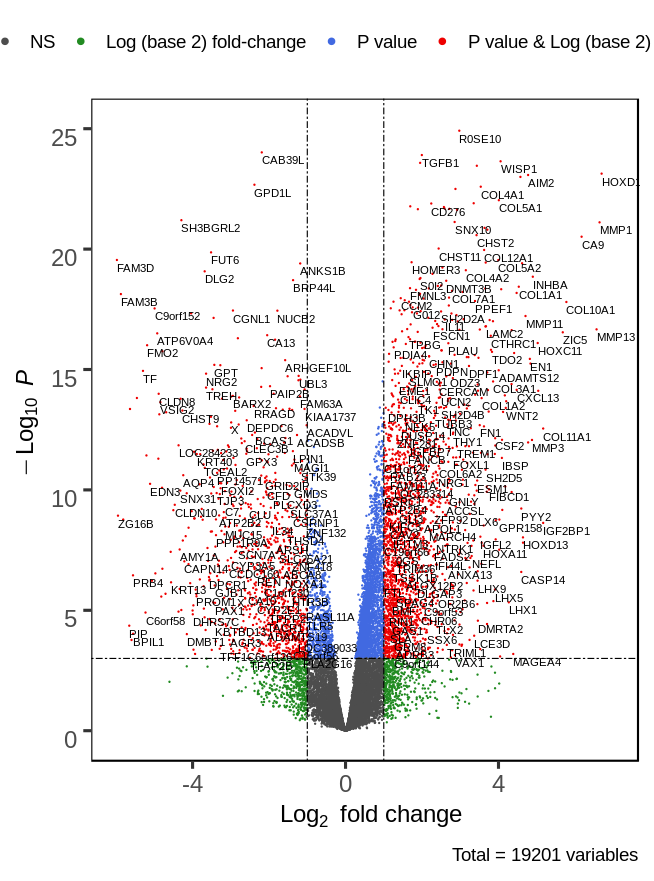

Supplement: Supplemental Information 18 — The significantly differentially expressed (|log2fc ≥ 1|p-value ≤0.05) mRNAs are shown as red dots. A total of 3831 mRNAs were found to be differentially expressed (2407 over and 1424 underexpressed). [file peerj-08-9656-s018.png]

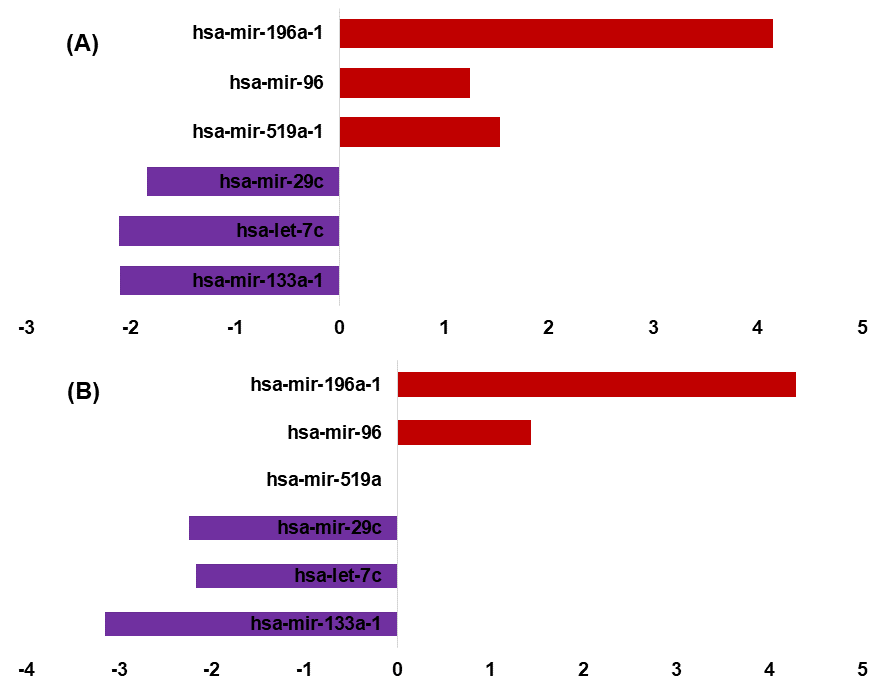

Supplement: Supplemental Information 19 — Differential expression of signature miRNAs in early (A) and late-stage (B) (|log2FC ≥ 1| p-value ≤ 0.05). In early-stage (miR-196a, miR-96) are overexpressed in early and late while (miR-29c,let-7c and 133a-1) are underexpressed in both early and late stage. The miR-519a is only overexpressed in early stage. [file peerj-08-9656-s019.png]

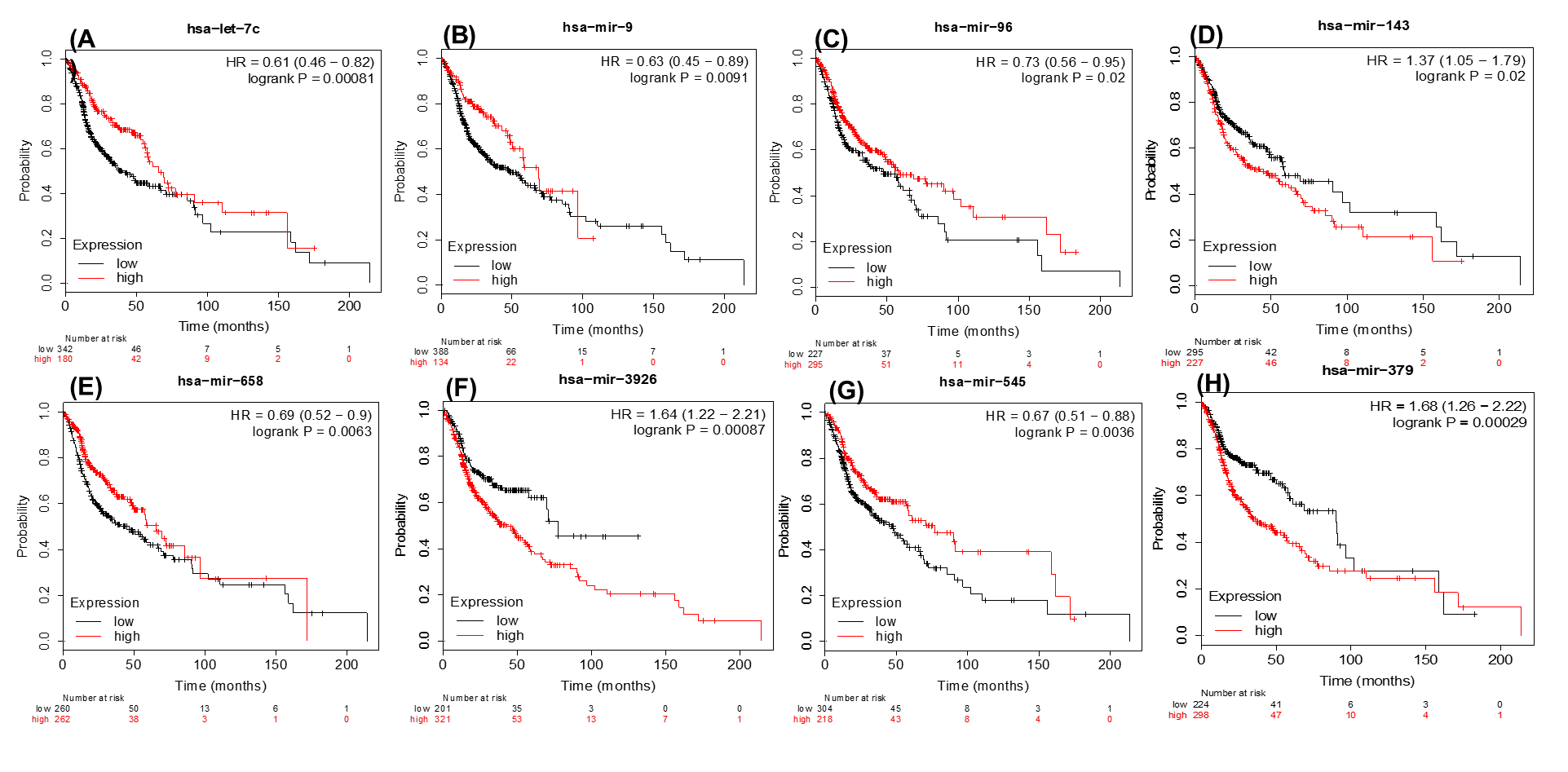

Supplement: Supplemental Information 20 — The low and high expression was classified based on the median value of the expression. The plots were generated from the KM plotter (p-value ≤ 0.05). The let-7c, miR-9, miR-96, miR-658, and miR-545 are positively poor survival correlated with overexpression of the patients while miR-143,miR-3926 and miR-379 are negatively correlated. [file peerj-08-9656-s020.png]

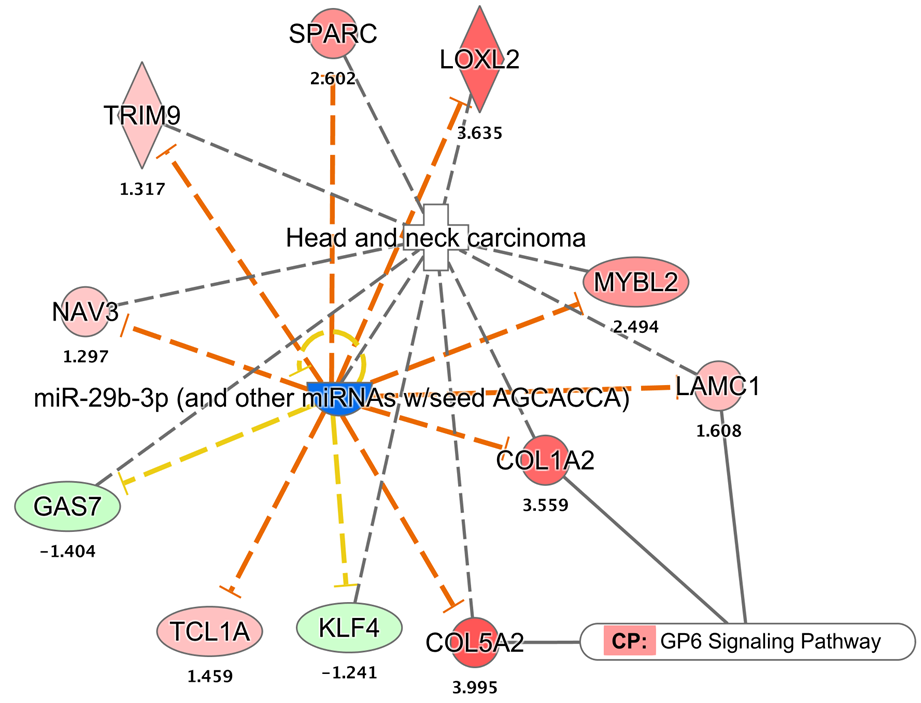

Supplement: Supplemental Information 21 — The miR-29c is a potential biomarker under-expressed in HNSCC. There are nine of its target mRNAs (out of 11) were found to be over-expressed in HNSCC. [file peerj-08-9656-s021.png]

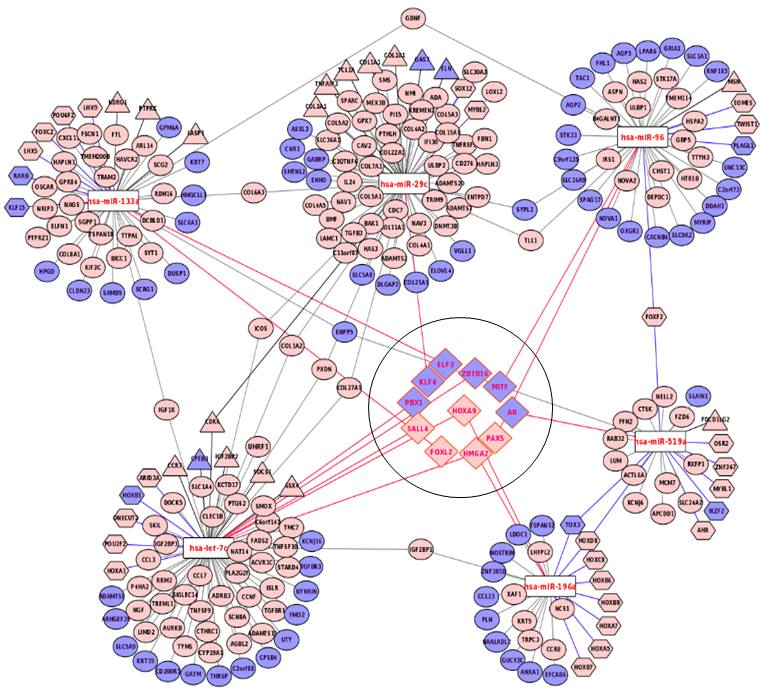

Supplement: Supplemental Information 22 — The mRNAs in light pink circle are over-expressed whereas those in the light blue are under-expressed. The let-7c has a number of targets comprised of the transcription factors (shown in hexagonal shape, N = 31) and cancer catalogue genes (shown in triangle shape, N = 19). The genes which are both transcription factors and are cancer census genes are shown in diamond. [file peerj-08-9656-s022.png]

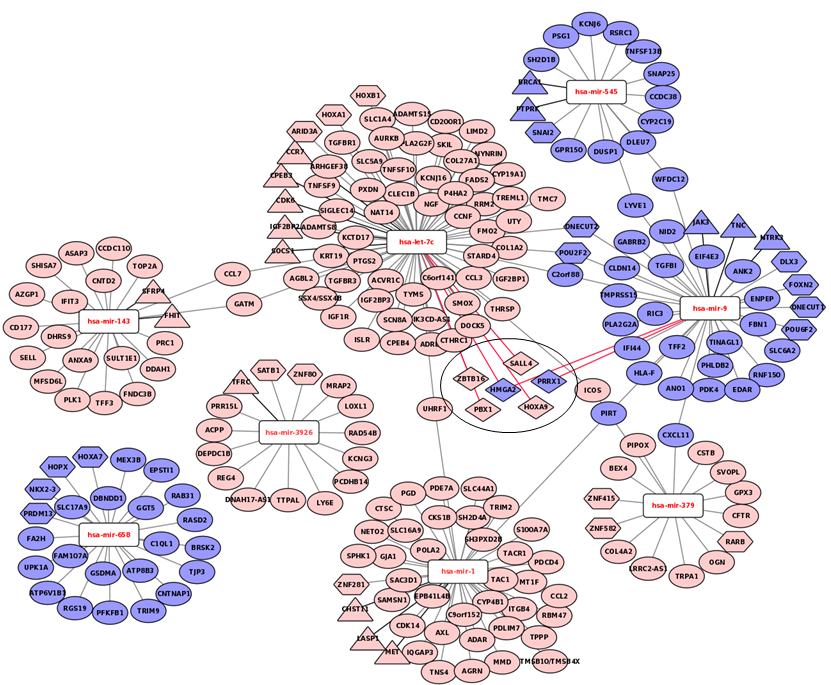

Supplement: Supplemental Information 23 — The over-expressed mRNAs are represented in light pink and under-expressed are shown in light blue. The let-7c, miR-143, miR-3926, and miR-1 were found underexpresseed while majority of their mRNA targets are found over-expressed. Similarly, the miR-658, miR-9 and miR-545 were found up and their targets were found under-expressed. There are 20 transcription factors among the targets while 16 mRNAs are on cancer gene panels. Six mRNAs are common among the transcription factors and cancer census genes. [file peerj-08-9656-s023.png]

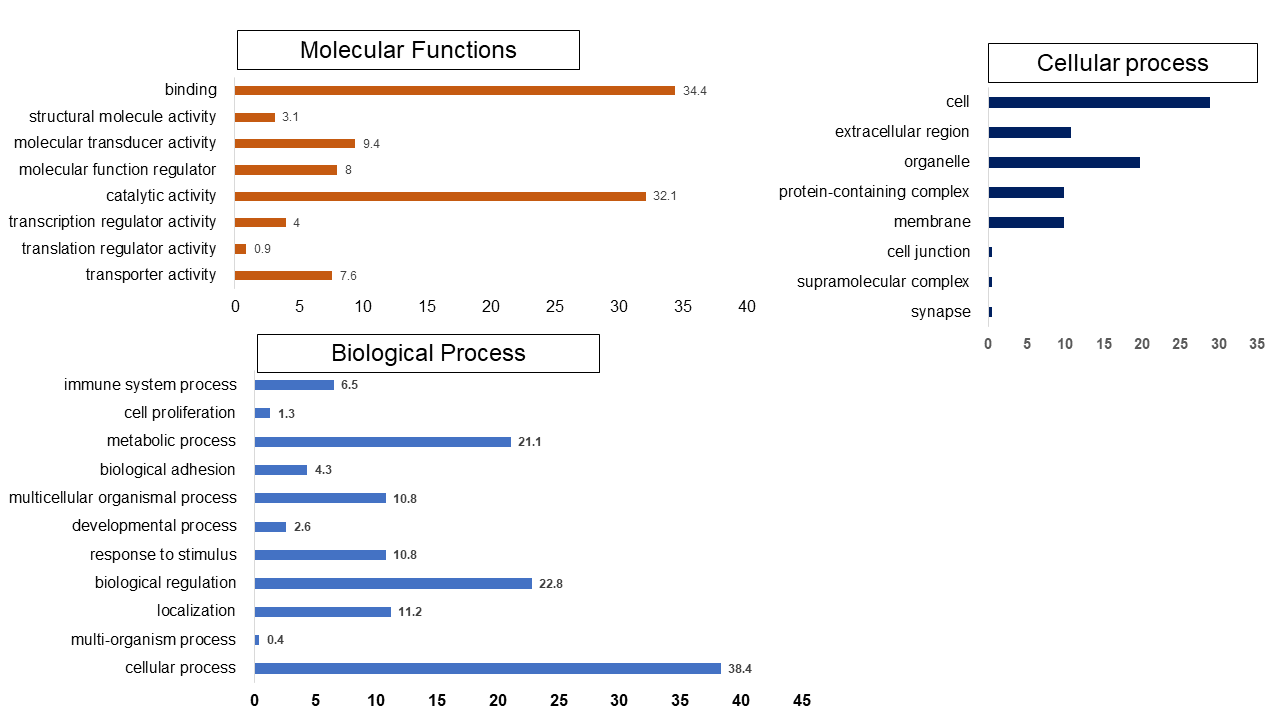

Supplement: Supplemental Information 24 — The binding and catalytic activity were overrepresented in molecular functions class. In molecular functions, the binding and catalytic activity of the protein is most enriched while the translation of regulatory activity is least. The cellular process and metabolic process were the most enriched biological process, while the cell and organelle with extracellular region were the top mapped cellular process. [file peerj-08-9656-s024.png]

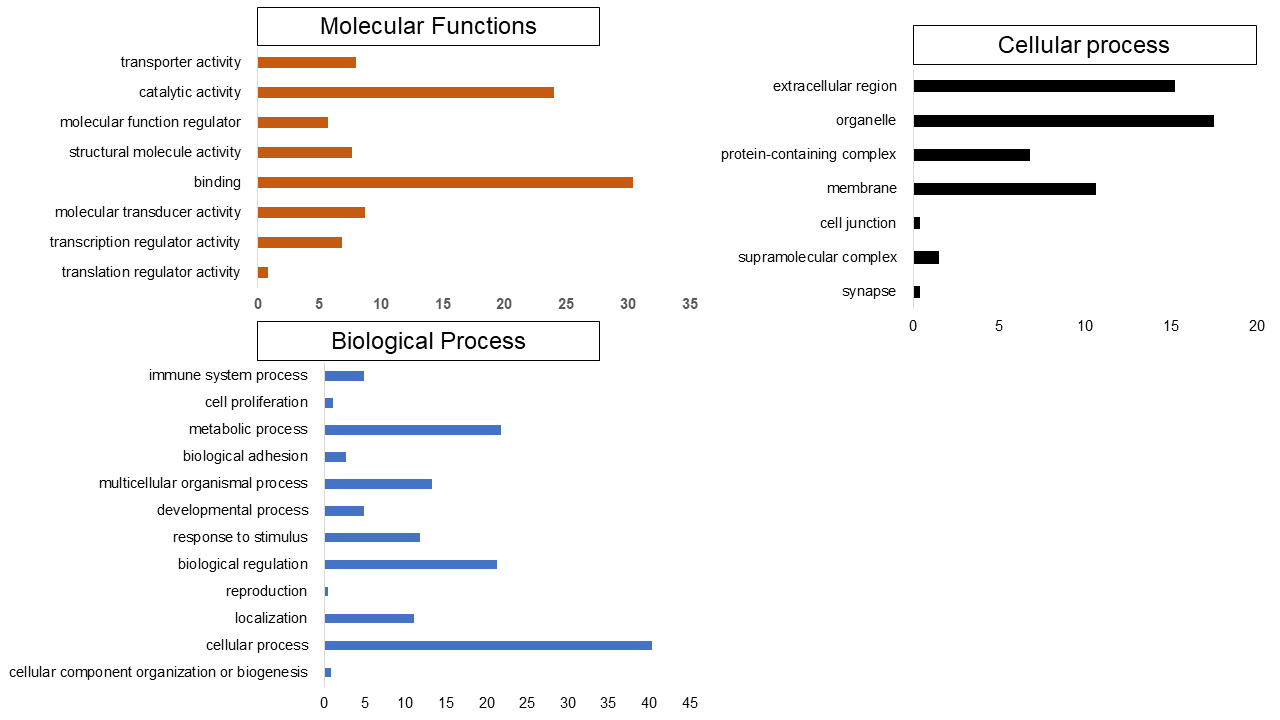

Supplement: Supplemental Information 25 — The enriched GO process were found to be quite similar as of the diagnostics markers as described earlier. [file peerj-08-9656-s025.png]
